# Lawson Wilkins and my life: part 1

**DOI:** 10.1186/1687-9856-2014-S1-S2

**Published:** 2014-05-28

**Authors:** Claude J Migeon

**Affiliations:** 1 502 Somerset Rd, Baltimore, MD 21210, USA

## Preamble

Several tributes to Lawson Wilkins have been published. They include, but are not limited to:

- “To Honor Lawson Wilkins in His 65^th^ Year.” Edwards A. Park and Alfred M. Bongiovanni. The Journal of Pediatrics Vol.57, Number 3. 1957.

- “Presentation of the John Howland Medal and Award.” Alfred M. Bongiovanni. The Journal of Pediatrics Vol.60, Number 4. 1960.

- “Acceptance of the Howland Award.” Wilkins L.

- **The Diagnosis and Treatment of Endocrine Disorders in Childhood and Adolescence**. Charles C Thomas Publishers. Springfield, Illinois.

○ Third Edition: John Money, Ph.D. 1965

○ Fourth Edition: Robert M. Blizzard, M.D. and Andrea Prader, M.D. 1994

- “Meeting Lawson Wilkins.” Alfred Jost. The Johns Hopkins Medical Journal 130: 38. 1972.

- “Pediatric Profiles: Lawson Wilkins (1894-1963).” Robert M. Blizzard. The Journal of Pediatrics. Vol.133. pg 577-580. October 1998.

- “The Early History of Pediatric Endocrinology.” Delbert A. Fisher, M.D. and Zvi Laron, M.D. Pediatric Endocrinology Reviews 1: 1. September 2003.

- Delbert A. Fisher. Pediatric Research 55. pg 715. 2004.

However, the postdoctoral fellows of Lawson and some colleagues felt that something more substantial should be written by those who trained with him. In 2004, some of us met in Baltimore. A program of chapters to be covered by each of us was prepared. This effort was supported by Dr. Ann Johanson with the help of the Genentech Co. Unfortunately, it appears that none of us kept our promises and nothing constructive came out of it.

Dr. William Cleveland was anxious to make a report of the history of the Lawson Wilkins Pediatric Endocrine Society and of the beginning of the field throughout the United States. This also did not come to fruition due in part to the death of William.

The project was then taken up by Judson Van Wyk, but sadly it was cut short by Jud’s death.

At this point in time, Betsy Wilkins McMaster and I decided that we should try again. We collected the papers of Jud Van Wyk and William Cleveland as well as old photographs that Barbara Migeon scanned into her computer.

Eventually, we decided that Betsy would write about the life of her father and mother. I would write about Dr. Wilkins and my life, as Lawson had been not only a mentor but also a father figure.

The present book is what came after two years of writing. My devotion, my admiration, my love for the man is very personal. However, I expect that the other fellows will identify with these feelings.

I also am writing for the next generation of pediatric endocrinologists so that they might know what a great mentor is and how to contribute to our field.

## Additional note

For several years, funds had been collected from past fellows and friends of Lawson Wilkins. With the help of Dr. George Dover, “The Lawson Wilkins Professorship of Pediatric Endocrinology” was established. The inaugural recipient was Dr. Sally Radovick on December 6, 2006.

## Introduction

From the first day we met, in 1950, I addressed you as Dr. Wilkins. This, of course, was a mark of respect that is characteristic of the European culture. In later years, some of the fellows of my age or even younger called you Lawson; I never could do this because of the enormous respect I always had for you. Even when I came to see you at Hopkins on the last day of your life, I asked, “How are you doing, Dr. Wilkins?” I hope that you will understand that it is out of respect and not lack of affection if I continue in this chapter to address you as Dr. Wilkins.

Clearly this memoir is about you, Dr. Wilkins, but starting in 1950, you have played such a major role at the most important junctions of my life that it is hard for me to separate my life from yours. First in 1950, you were willing to bring me to the Johns Hopkins Hospital. Then in 1952, when I had to make a decision on what to do next after two years of fellowship, you convinced me not only that I should come back to the United States but also that I should enlarge my field of knowledge by going to another center in order to learn more basic science. For that purpose you wrote to many of your friends about available post-doctoral fellowships and helped me choose among them. This is when I went to work at the University of Utah in Salt Lake City. In 1955, when I was ready to leave Salt Lake City and was planning to join John Crigler in Boston at the Children’s Hospital, you gave me a call along with a letter asking me to come back to Baltimore as an Assistant Professor. And, of course, I did. In the following years after my return to Baltimore, you hinted on many occasions about my getting married. You even made some suggestions. Eventually, when I married Barbara in 1960, you were one of the wedding party of five. Later when I considered moving to the University of Florida in Gainesville, you asked Bill Thomas to give you a little time to figure out how you could keep me in Baltimore. And there again I did what you suggested. So I usually find it difficult to talk about Lawson Wilkins without recounting my life and career.

## University of Paris, School of Medicine (1949)

My eagerness to visit the United States initially arose from a rich personal experience.

**Figure 1:**
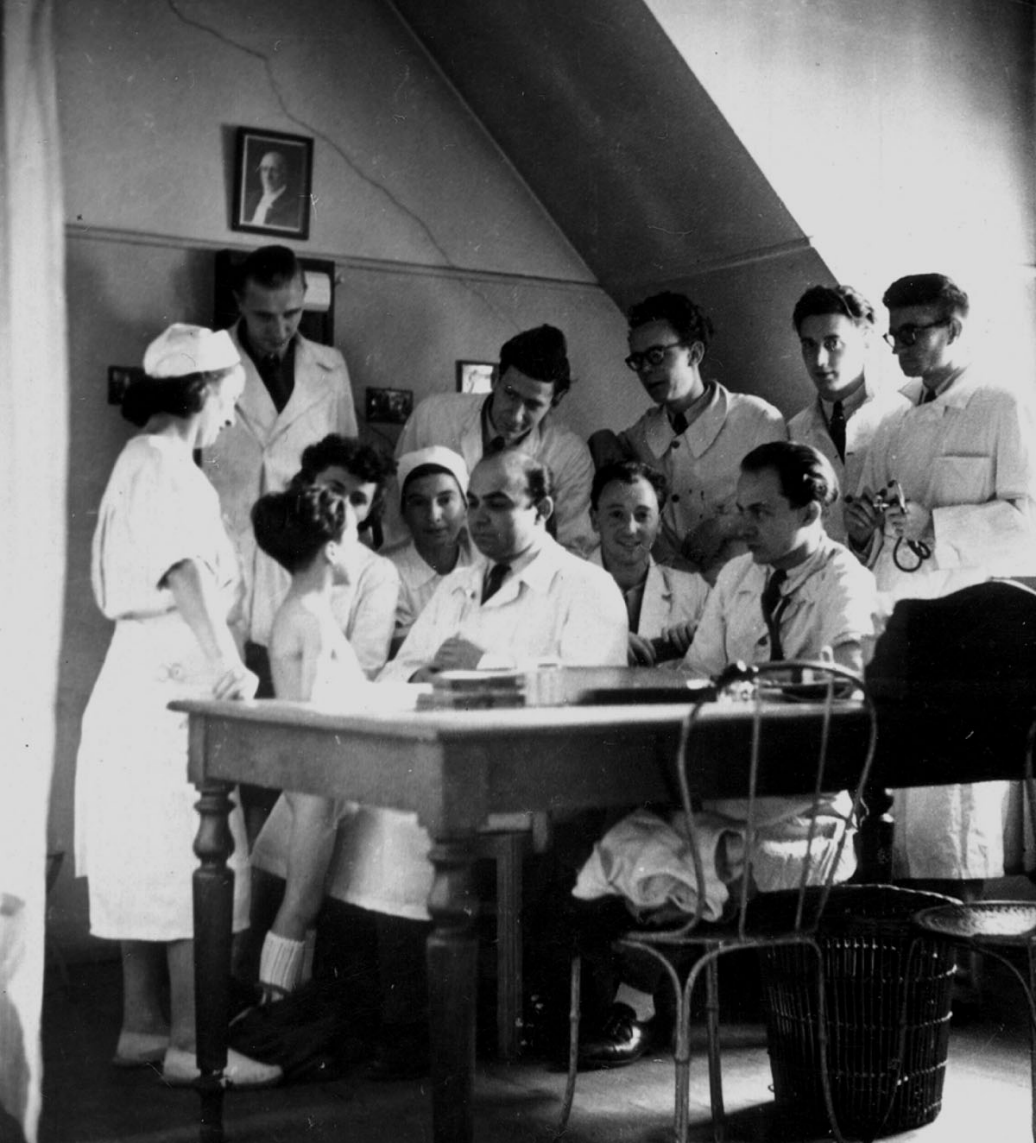
The “consultation externe” at the Hopital des Enfants Malades. (1948)

In 1949, there was only one medical school in Paris, the University of Paris. However, the clinical clerkships could be taken in all the hospitals around the city. I chose the Hopital des Enfants Malades in Necker. There, I worked with Professor Julien Huber and Professor Sotirios Briskas. (Figure 1) Indeed, I did my medical thesis in that department. Dr. Briskas had a sister in Chicago who had two sons and a daughter. During the Second World War, both sons were in the armed forces. Both of them came to France; the oldest was killed in the Battle of the Bulge in Bastogne. At the time of this battle, I was in Rethel (Ardennes) about 50 miles south of Bastogne. I could hear the deep rolling noise of the artillery and it was quite frightening.

His brother and sister, William and Stella Nanos, decided to visit their uncle in Paris and also to go to the grave of their brother who was in the large American Cemetery of Bastogne. Dr. Briskas had asked me to help entertain them. We eventually visited their brother’s grave. We drove there with my family. This was a powerfully emotional experience for them and for me. Of course it brought us closer together and during their nine months in Paris, we were extremely close. The day they left was a sad day for all of us and we promised to see each other again in the future.

This is how the idea came to me that I should try to obtain a fellowship in the United States. I applied at various institutions. In addition to my medical studies, I had obtained certificates in chemistry and biochemistry. As part of this work, I had done some research about the effects of copper on metabolism of rats. Having seen a flyer on the bulletin board at the School of Medicine, I applied for and received a Fulbright Fellowship. However this fellowship did not require that I have a specific place to work. During my interview, I suggested Chicago would be a good place to work. However, somehow with my background and with my clinical interest I was assigned to the Pediatric Endocrine Clinic of Dr. Lawson Wilkins at the Johns Hopkins Hospital.

I was extremely pleased with this assignment, although I would have preferred to be in Chicago closer to my friends. When I told Bill and Stella of the location for my fellowship, they suggested I could easily visit them at vacation times.

My knowledge of geography was quite good, and I could envision Washington, Philadelphia and Boston on the East Coast of the United States, but I did not know where Baltimore was located, until I consulted an atlas and learned that Baltimore was very close to Washington DC.

## In Paris (Spring 1950)

I received an official typewritten letter from Baltimore, signed by Lawson Wilkins. I was quite pleased. In my clinic at the Enfants Malades, I had seen patients with Turner Syndrome and had read papers written by Wilkins. I never learned why I was accepted. My medical thesis was entitled: “L’ossification: influences endocriniennes. Importance en thérapeutique infantile.” The thesis even got a silver medal! Dr. Wilkins must have thought that I was interested in learning more about the endocrine effects on bone growth. He had an NIH grant titled “Relationship of hormones on growth and development.” (AM-00180). In his letter, Dr. Wilkins announced that he was going to be in Paris, stopping on his way to the Sixth International Congress of Pediatrics which was going to take place in Zurich, Switzerland. In addition, Dr. Wilkins asked to meet with me, if possible, at his hotel.

Needless to say, I was excited as well as nervous about this first encounter with Dr. Wilkins. I’m not quite sure that I knew what to expect but for some reason, despite the letter of acceptance, I had the feeling it was going to be a test which might influence whether I really got a fellowship and the position.

## Meeting the Wilkins Family

**Figure 2:**
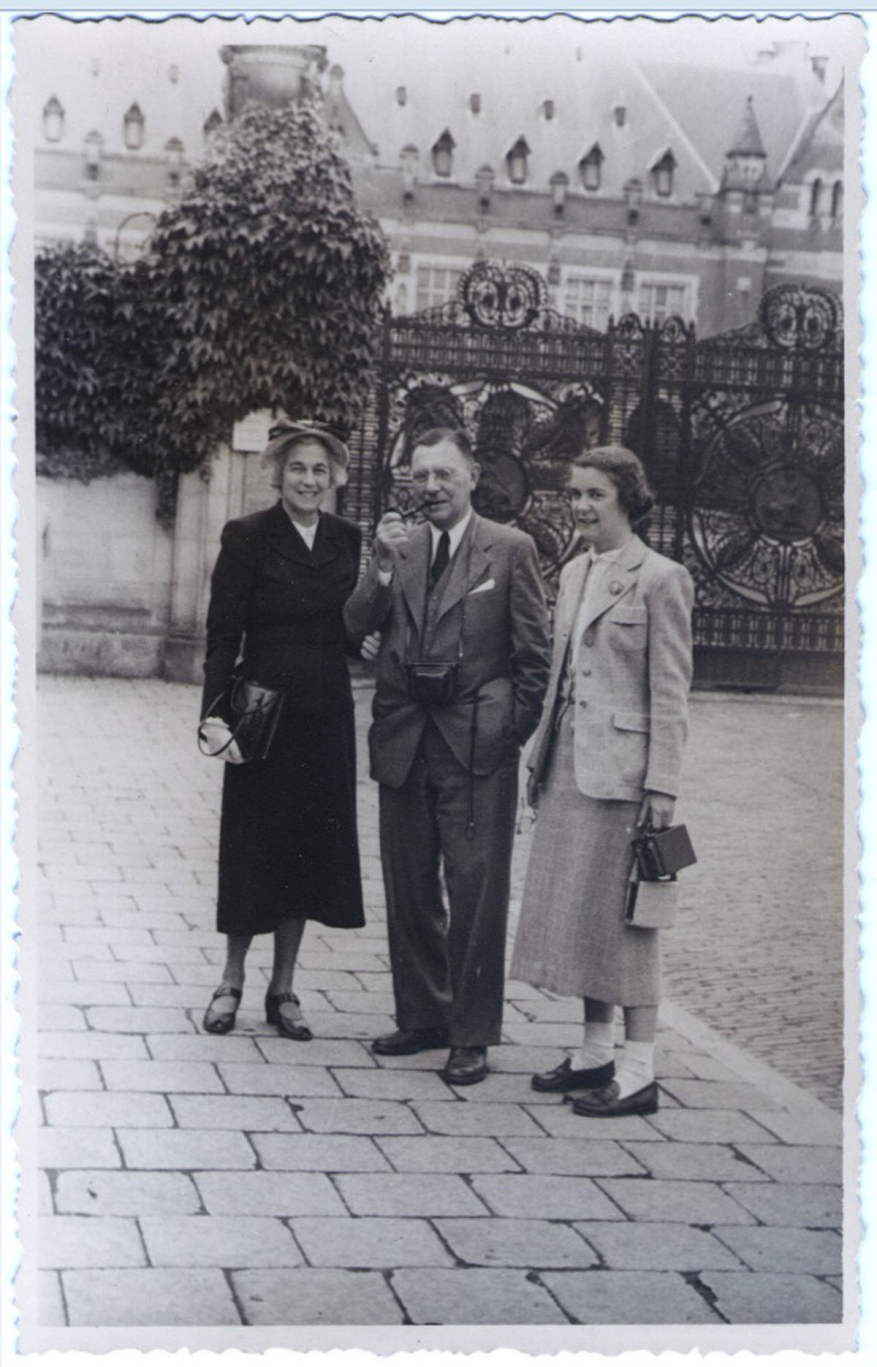
Lawson, Lucile and Betsy Wilkins, The Hague, 1950

On the chosen day, I took the subway from the hospital to the hotel where the Wilkinses were staying. I must have been a bit late because, when I arrived to the hotel, there were three people lined up waiting for me on the sidewalk, starting from the right, Dr. Wilkins, and then Mrs. Wilkins and their teenage daughter Betsy. (Figure 2) Perhaps because they were standing on the sidewalk and I was at the level of the street, they appeared to me to be quite tall. Later, I realized they were average height. Dr. Wilkins wore his usual gray charcoal black suit with a button-down blue shirt and a dark blue tie. He looked intimidating to me, a cigarette in his right hand. Mrs. Wilkins was quite elegant and very fashionable. And then there was a teenage girl, who was introduced by Dr. Wilkins as his daughter, Betsy. She appeared to be a young lady who knew the world. On the other hand, I felt rather ignorant of the proper manners for such encounters.

Dr. Wilkins directed the flow of questions, but I have to admit I did not always understand what he said, because at this time in my life, I certainly didn’t have a deep knowledge of the English language. I’m not sure if Dr. Wilkins said that he was accepting me or was pleased to meet me, it was not clear what he said.

Eventually, I understood a very specific question from Mrs. Wilkins: where could she purchase gloves? I believe that she added that France was well-known for the quality of gloves and that she was anxious to find a place to buy such gloves. I have to admit I had no idea where she should go to find gloves. My own gloves were bought by my mother, and I had no idea where she purchased them. So I had to say that I was sorry I did not know. I really felt terrible, and so incompetent, not knowing where somebody coming from the United States could find the best quality French gloves.

I believe the next question was from Dr. Wilkins, asking me where there was a good restaurant that would not be too expensive. I believe that is what he asked. As a medical student I was not very affluent. Toward the end of medical school and before passing the final thesis, one can replace a vacationing physician at least for a summer. I had done that a few times. This was my source of revenue for the rest of the year. I was also in the business of giving intravenous injections as a treatment for venereal disease to foreigners in their hotel rooms, per orders of Dr. Briskas. It seemed to me that most of them needed therapy because they had a positive syphilis test. Penicillin was not “invented” yet, and intravenous arsenic therapy was the treatment of choice. Neither of these activities made me very rich and I certainly never went to restaurants. So, during this interview I felt trapped for a second time, unable to answer questions of interest to the Wilkins family. I remember breaking into a cold sweat, believing that I had totally blown my chances of being accepted by Dr. Wilkins for my fellowship. Reaching very deep in my memory, I retrieved the name of a restaurant. Although I had never eaten there, I said “Well, maybe Le Grand Vefour.” I remembered that name because it was unusual, but I didn’t know that it was, indeed, a high-class restaurant, which certainly was very expensive. I doubt that the Wilkins family ever went there, and I never returned to this conversation to find out.

By that time, I had the feeling that the two ladies, aware of my anxiety and feelings of incompetence, decided to return toward the entrance of the hotel. Dr. Wilkins tried to tell me that he was going to make an important presentation in Zurich. Our only difficulty was that we were having a two-way conversation, he not understanding too well what I said in poor English, and I being able to understand only parts of his comments. After a few minutes of this futile exercise, we said goodbye. I went away from our encounter totally deconstructed, as the philosophers would say. Shaking hands with Dr. Wilkins, I was miserable, feeling that I had ruined my chances of going to the United States. I went to a bistro and asked for a café creme. Feeling terribly cold inside, I don’t know if it was the weather or the remnants of that cold sweat. I was so unhappy about my inadequate performance.

Betsy McMaster has also written about this encounter and the question of whether Dr. Wilkins could bring his daughter to the Folies Bergéres. I do not have a clear memory of this part of the conversation. However, I have to state that Betsy greatly exaggerated my early age going to this spectacle. I was not five years old; actually I was six years. My parents had bought tickets for the Promenade (the space behind the boxes, where you must stand up to see the show). The trouble with the Promenade is that it is separated from the boxes by a tall wooden partition. My father had to lift me to see the stage. But when we noticed that a box was empty, I sneaked down there and had the best possible seat: I very much enjoyed the show!

## June - July 1950

Despite my first catastrophic interview with Dr. Wilkins, I continued to fill out a number of papers which were requested for the actual fellowship application. I obtained letters of recommendation from my clinical chiefs, Drs. Julien Huber and Sotirios Briskas, and Professor Michel Polonovski who was head of the Department of Biochemistry at the Medical School, with whom I had been doing some research during the previous two years.

At the same time I had to finish putting together my medical thesis. The text had been typed by a secretary and figures had been made to go along with the text. All this material had to be assembled and a total of fifteen copies of the thesis had to be delivered to Ecole de Médecine.

In addition, not knowing whether I was actually going to obtain a fellowship, I had to make plans for my financial support. I had made arrangements to go to Lievin in the Pas-de-Calais (my actual birthplace), where I would stand-in for a vacationing pediatrician from June 30^th^ to August 3^rd^.

During that time I was too busy with patients to think about my fellowship with Dr. Wilkins.

## On to Johns Hopkins Hospital! (August 1950)

When I came back to Paris on August 4^th^ there was a letter asking me to appear at the Service Universitaire des Relations avec l’Etranger, 55 Rue St. Dominique on August 9^th^ at 2 p.m. This interview went well, even though it was carried out in English. I was told to see Mademoiselle Papillon (no joke!) on Saturday the 12^th^ to apply for my passport and US visa. While picking up my passport on Friday the 18^th^, I met a young lady, Mademoiselle Nadine Gregouroff, who was also an applicant for a Fulbright Fellowship. We tried to reassure each other about this great adventure that we were embarking on. She mentioned that she had met a few other French students who were also going. She had an appointment at the American Embassy for her visa on the 24^th^ of August. Mine was the next day, on the 25^th^. I met with Mr. Holt at the American Embassy who carried out all the appropriate formalities and gave me my visa. Mr. Holt was very kind. He explained that after arriving in New York, I would go to Washington, DC where I would spend four weeks getting accustomed to the American way of life and to the language (someone had probably noted my poor English). Then, I would take the train to Baltimore.

Finally he told me that my boat would depart on August 31^st^, six days later. Mr. Holt stated that he hoped the visit would be educational and profitable. At the door of his office, he shook my hand, gave me a pat on the back, wishing me a good trip - Bon Voyage - and good luck.

## The trip to the United States of America

Abruptly, it dawned on me that I was really departing for a long trip across the Atlantic and that I had to prepare for it. During the six days that remained, I had to go and get my tickets for the train and boat from a travel agency. I also had to obtain the addresses of my contacts in Washington, DC and in Baltimore. This was a most stressful six days, saying goodbye to friends and family, finding a place to store my books and belongings. There was also the need to make a trip to the bank to determine my real worth. I knew that the end point was the **Harriet Lane Home**. To go to “home” seemed quite propitious to me. The French people think of “home” as a “homey place” with a congenial environment. At that time, I did not know the full name of the Department of Pediatrics at Johns Hopkins Hospital: The Harriet Lane Home for Invalid Children.

**Figure 3:**
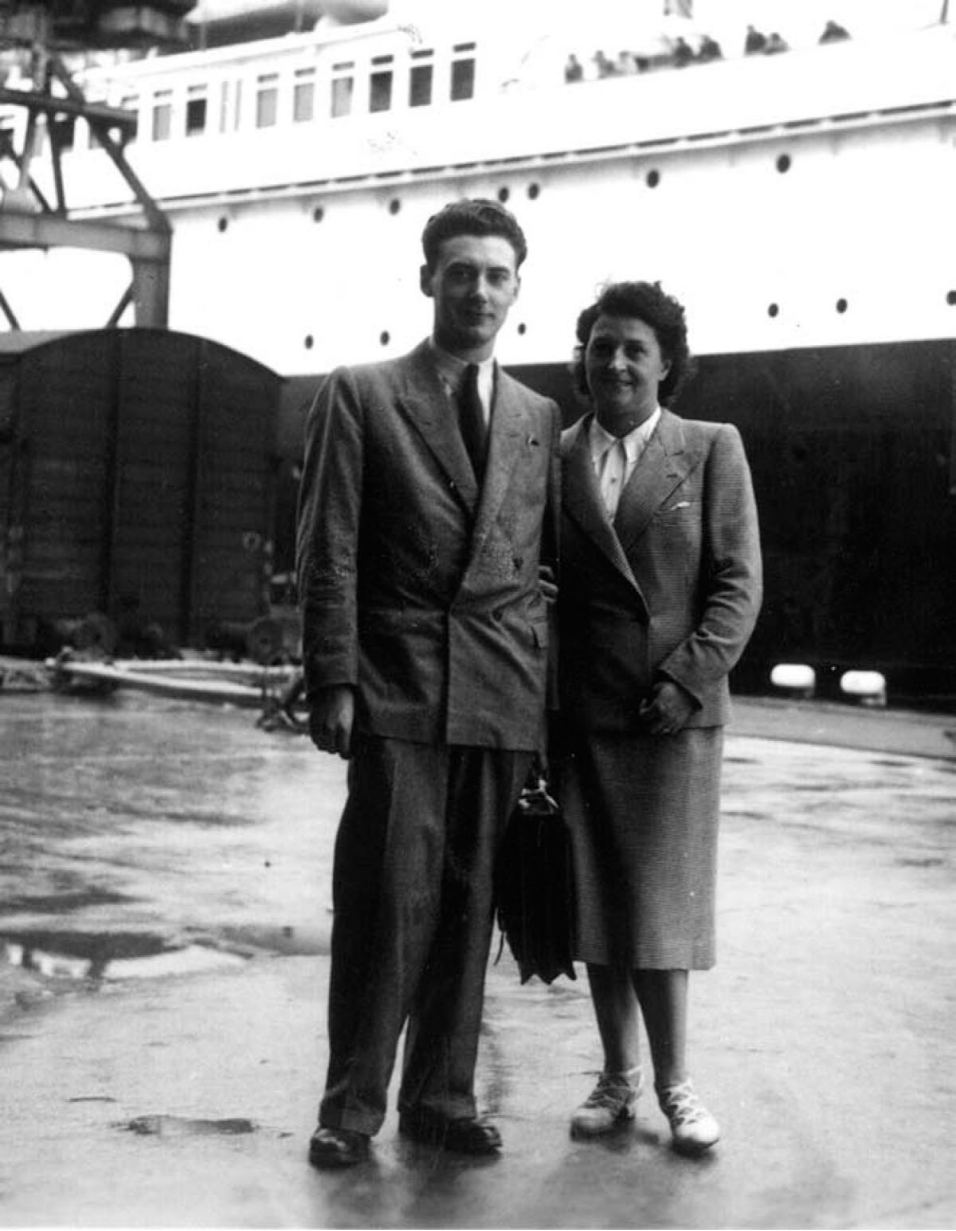
My aunt (adoptive mother) as I get ready to embark on the De Grasse at Le Havre. (August 30, 1950)

Thursday, August 31^st^, 1950: that was “Departing Day”. I had to be at the station at 9:22 A.M. for a train to Le Havre. It arrived at noon. I carried a large suitcase and a raincoat full of hope in the pockets. When I arrived in Le Havre, my family had driven there with my brother Michel and sister Claudine, all of them saying goodbye and shedding a few tears. (Figure 3) I boarded the boat and waved goodbye to France. It was certainly a very poignant moment when I saw my family disappearing slowly in the harbor as I was moving away from the pier. However, I cheered up after a few hours as I met the other Fulbright Fellows who were going to the States. Like me, they were sad to leave their families but also excited to go. We kept each other company, while discovering where each of us was going. One was headed for Philadelphia, another to Indiana and another to Seattle. One of them was to stay in New York. There was also a minister who was joining a religious school in the South. Needless to say, we tried to reassure each other and actually had a wonderful time discussing our past and our plans for the future. In those days, travel by boat was an adventure in itself. First we went to Southampton where we stopped for several hours, and nine days later on Saturday, September 9^th^ we arrived in New York at 8:30 P.M. The view of the lighted Statue of Liberty was incredible. Because it was late, we could not disembark and we had to wait until the next morning, Sunday. Perhaps because we were a little bit scared of being on our own, we remained together all day Sunday. On Monday, we separated and headed off in our individual directions.

## Washington DC: preparing for Johns Hopkins (September 1950)

My orders were to go to Washington. At the address given to me, a secretary told me that I had a reservation in a boarding house on 1406 10^th^ Street NW. All the other boarders were American; about fifteen of them. None were Fulbright Fellows. They were in Washington for a short period of training or study. During our suppers together they introduced me to a new way of life. When corn on the cob was served, as a well-educated Frenchman, I approached the cob with my fork and knife; somehow the cob slid off my plate and ended up in the middle of the dining table. That was good for a laugh! Dr. Wilkins also laughed when I told him about it and it became part of the repertoire of my misadventures. I also told him about my first experience with baseball. One night, the boarders encouraged me to go to the game with them. I explained that I was not familiar with baseball but the boarders assured me that they would teach me as the game progressed. Afterward, my perception was of long periods of inaction (eating hot dogs and drinking beer) interrupted by a few periods when all the spectators were standing, gesticulating and shouting. There was no need for explanation during the quiet periods and I could not get any explanation during the rare periods of exaltation.

Dr. Wilkins pointed out that many words of the baseball language were often used in everyday conversation. And so I learned about “a hit,” “home plate,” “home run,” “first base,” etc.

The days in Washington were quite busy and instructive. It was a wonderful experience with visits to museums, the Senate and the House, and the White House. And, of course, I saw all the great monuments, including the imposing statue of Lincoln and the Jefferson Memorial. There were also well-organized lectures on the pronunciation of the English language. I worked very hard at this, as I was anxious to make a better impression on Dr. Wilkins at our next encounter.

## At Johns Hopkins Hospital, under the dome (October 1950)

**Figure 4:**
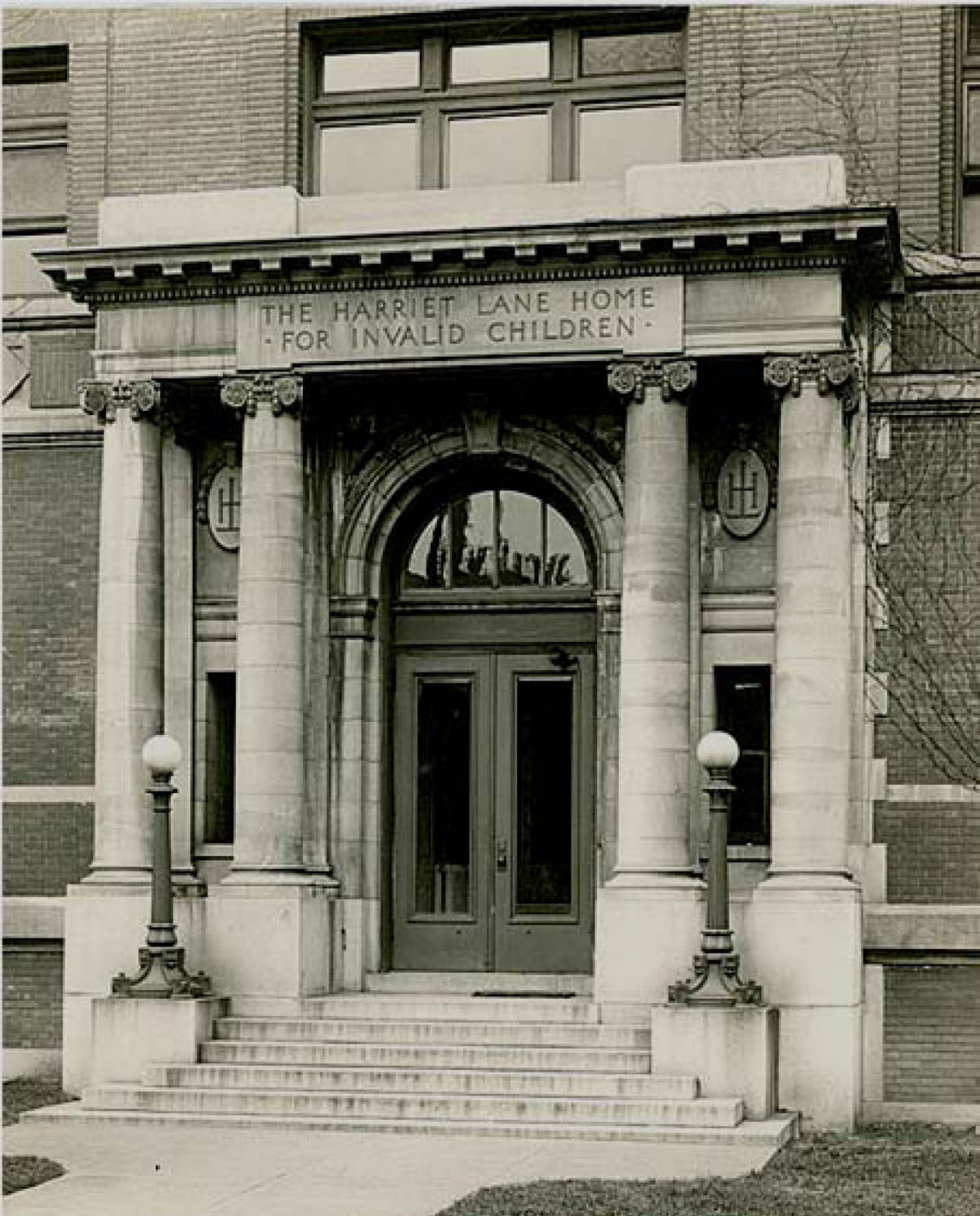
The Harriet Lane Home. (October 1950)

On Monday, October 9^th^ I re-packed my luggage and took a taxi to Union Station on my way to Baltimore. Another taxi took me from Penn Station to the Johns Hopkins Hospital. The driver deposited me at the entrance on Broadway and helped me with my luggage. As he turned to me, the driver must have noticed some doubt on my face. Pointing to the main entrance, he said, “This is it!” I looked at the Dome and the 19^th^ century main building with its two annexes. It looked quite old and old-fashioned rather than modern, as I expected. It was a terribly hot day, 90 degrees or more at noontime. I picked up my luggage, climbed the steps to the entrance of the hospital and was met by the doorman who looked at me with a somewhat suspicious eye, wondering where I was going with my large valise. I told him that I was going to the Harriet Lane Home. (Figure 4) Certainly; he explained how to go there, but I did not understand everything he said. I moved inside, put my luggage down and raised my eyes to see the bigger-than-life statue of Jesus Christ in the entrance hall under the Dome. I sensed that he felt sorry for me; or was it that I felt sorry for myself? At least it was cooler under the Dome. After some rest, I picked up my luggage again, moved around the big stairs, turned right in the corridor and then left – finally, arriving in a building that I was told was the Harriet Lane Home.

I was most disappointed: in my imagination, I had visualized a beautiful, pleasant “home”. The first floor of the Harriet Lane was far from this: rather old, small, very busy. At noon, everybody seemed to rush around and talk very loudly. Somebody again asked me where I was going and I explained I was trying to find Dr. Wilkins. I was told to take the elevator and go to the 5^th^ floor. The elevator was easy to locate. This was a big double-door of very shiny red copper. When the elevator came down, the two doors were opened by a “colored lady” (as it was politically correct to say then). She helped me get my luggage into the elevator and told me that her name was Odessa. She was a wonderful young lady, always smiling. Her job was to open and close the two doors of the elevator, and once they were closed, to maneuver a lever to bring the elevator up or down. It was always a problem to stop exactly at the right level. Sometimes Odessa would miss the floor and would have to close the doors again and run the elevator down a little bit to make it just at the right level. We reached the 5^th^ floor and again Odessa helped me with my luggage.

## The 5^th^ floor of Harriet Lane Home

I guess I was getting tired by this time. Also I was overtaken by the heat as the 5^th^ floor was directly under a flat roof. I had no means to check the temperature, but I suspect it was at least a hundred degrees. This 5^th^ floor did not ease my disappointment with my new “Home”. The corridors were cluttered; there were several cases of bottles of Coca-Cola, drums of organic solvents; some appeared full, others empty. There was a pile of serving trays on the floor. A small table near the elevator was holding a half dozen bottles containing yellow liquid –probably urine samples brought up from the clinical floors by a nurse.

Again someone asked me where I was going, and I explained that I wanted to find Dr. Wilkins. “Go to the end of the corridor and then turn right and you will find his office right there.”

Straight ahead at the end of the corridor a door opened to an office with typewriters and secretaries. On the right, I found a narrow, short passageway; on one side, the wall was covered with shelves from the floor to the ceiling. Those shelves were loaded with jars of chemicals. I’m sure there were more than several hundred bottles sitting there, with all sorts of sodium and potassium salts, including potassium cyanide. And then I came to the office of Dr. Wilkins. I knocked at the door which was actually open. He was sitting behind his desk, a cigarette in his hand. Noticing that I carried a big piece of luggage, he suggested that I leave it in the corridor as there was not that much space in his office. Indeed, besides two shelves with books, his chair on one side of his desk and my chair on the other, there was certainly no space for luggage. He welcomed me very warmly. With his very deep bass voice, he asked whether I had had a good trip, and I, of course, returned the compliment, asking if he had a good visit in Switzerland. He reported that his exhibit in Zurich had been extremely well-received. He also explained that the exhibit was a summary of his book which had just been published (The Diagnosis and Treatment of Endocrine Disorders in Childhood and Adolescence. First Edition. 1950). He had received a few copies and he presented me with one of them that I could read it at my convenience. Dr. Wilkins took his pen and wrote on the front page: “To my good friend and colleague, Claude Migeon.” Later, when I checked his writing, I was amazed and warmed deeply in my heart about the word “friend.” I was also surprised and flattered to be referred to as the colleague of an American Professor who had just written a textbook which was to become the “bible” of pediatric endocrinologists.

He also brought up the question of lodging. He told me that the House Staff had rooms in various places, some were in the building called “Main Residence” which was near the Wilmer Institute; others had rooms under the Dome and at the Nurses Home across the street on Broadway. At that point, Dr. Wilkins got up from his chair and took me to meet everyone and to visit the laboratories. The labs were certainly close to his office since they were just next-door, at the end of the narrow corridor.

## The people in the labs of pediatric endocrinology and their close neighbors

As we entered through the door, someone stated that there was a lot of organic solvents being used and to please keep the cigarettes out. Dr. Wilkins agreed, made two steps backward and kept his lighted cigarette behind his back, like a child caught smoking by his parents.

This first lab was a very large room with a working bench in the middle, each side of the bench being a working area. There were also benches on three sides of the room. The fourth side, on our left when we entered included a big fume hood and on the right was a large sink attended by a short black lady of about 50 years of age, Miss Virginia who prepared glassware. She was the first person I met in the lab; I was told that she was a wonderful lady who was most important to all the work of the lab as all the results depended on the cleanliness of the glassware. Miss Virginia was born and raised in the Johns Hopkins neighborhood. She had worked all her life at the hospital and was part of a group of African-Americans who labored in this Institution and were thoroughly dedicated.

**Figure 5:**
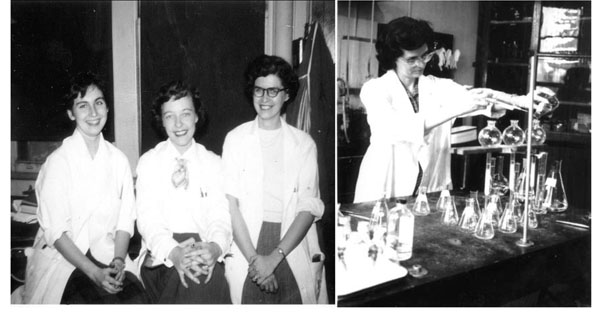
(a) The hardworking technicians. From left: Ruth Fleischmann, Marie Ellen Crofton, and Betty Lawrence. (b) Betty Lawrence running the columns to measure levels of 17-hydroxycorticosteroids in blood.

Then Dr. Wilkins introduced me to the two technicians, Henry Schulte and Mary Ellen Crafton. (Figure 5) They said hello rather quickly as they were very busy with their work. The last person in the room was Dr. Lytt Gardner. Dr. Wilkins explained that Lytt was an Assistant Professor. He had just arrived from Boston where he had been working with Nathan Talbot, Edna Sobel, Fred Bartter, Jack Crawford and Ann Forbes. At that time, I did not know these investigators, but the fact that Dr. Gardner was coming from Harvard was impressive enough for me. After the busy and noisy laboratory, Dr. Wilkins showed me two little rooms which were located on the other side of the galleries of chemicals. In the first one we stowed my luggage. In the other small room was a huge instrument taking almost all the space, and a young tall man, Dr. John Crigler. At that point, Dr. Wilkins told me that this instrument, a flame photometer, was the last word in electrolyte measurement. This was most important in the study of electrolyte balances in the children with salt-losing adrenal hyperplasia. I was also told that John had just finished his year as Chief Resident at Harriet Lane and that now he was deeply involved in the studies of electrolyte balance. A few bottles of urine were sitting on a cart and I guessed that the other bottles near the elevator would eventually end up here.

Out of this room and at the entrance of the narrow corridor were two pieces of furniture which did not appear to belong to each other. It was explained that one was a muffle furnace which went to a very high temperature in order to activate various earths needed for column chromatography. The other was a refrigerator which belonged to Dr. Harold Harrison. On one side of this hot/cold system was the secretaries’ office; Miss Mary Bachman and Mrs. Idaline Ratcliffe were the ladies who had “worked untiringly on the preparation of the Zurich exhibit and on the manuscript” of the Wilkins book. When Miss Bachman retired, she was succeeded by Miss Eleanor Warfield, a distant cousin of Mrs. Warfield-Simpson who married The Duke of Windsor. Dr. Wilkins was very solicitous of his secretaries, often giving them a ride home. I must note that Baltimore is quite proud of its relationship with the British royalty as well as the Bonaparte family.

A door on the other side of the icebox entered into the Harrisons’ laboratory. We went in and I met Dr. Helen Harrison who was a Ph.D. doctor, working on calcium metabolism. I was told that her husband was the head of Pediatrics at Baltimore City Hospital; he was coming to the lab in the afternoon, the morning work being carried out by his wife.

Near the elevator was another large laboratory. This was the domain of Dr. Hugh Joseph who was a hematologist; he had been at Harriet Lane for many years. Dr. Joseph was a very soft-spoken and very kind gentleman. After his welcome words, we established that his wife, Dr. McCarthy, had been driving American ambulances in 1917-1918 in the northern part of the Allied front, near my hometown of Lens (Pas-de-Calais) in France. Dr. Wilkins was quite amazed; he explained that, at that time, he too was in France with the Hopkins group.

Leaving Dr. Joseph’s lab, Dr. Wilkins passed the elevator and immediately we entered another lab that belonged to Endocrinology. On the left of that room, a low table was holding an instrument: a Geiger counter. Dr. Wilkins explained that the clinic used this counter to determine the radioactive iodine, ^131^I, thyroid uptake of our patients, and that it was a very useful new methodology. Apparently, we shared the cost of the ^131^I with Dr. Sam Asper in Adult Endocrinology. The radioactive iodine was sent directly to Hopkins from the Atomic Energy Commission. At the back of the room, in front of the windows, there was a high lab bench. Half of it was covered with papers belonging to Dr. John Crigler. Dr. Wilkins suggested that I could use the other half of the bench for reading and writing. Now I had an office!

Dr. Wilkins and I left this room but went no further: this was the limit of Pediatric Endocrinology. I was told that, at the other end of this building, were the laboratories of Dr. Victor Najjar and also a routine bacteriology lab for the pediatric patients, run by Mrs. Bonny Behner.

Dr. Wilkins concluded our tour, saying that this was all of the 5^th^ floor of Harriet Lane. This floor had been added after the first four patient floors had been constructed. He also explained that it was rather hot here because of the structure of the building. Needless to say that, in those days, there was no air-conditioning. I did not need Dr. Wilkins’ explanation for understanding that this place was the closest to hell one could ever go unless one was actually in hell itself.

Indeed, this was an absolutely miserable environment in which everybody suffered but no one appeared to complain, so it was difficult for me to even comment. However, I must confess that I suffered a great deal for 13 summers, until we moved to the new and air-conditioned Children’s Medical and Surgical Center in 1963.

## Student lodgings at Hopkins

After this orientation, Dr. Wilkins sent me to find lodging. I first went to the Nurses Home on Broadway. The lady at the desk laughed at me: she explained that men were not permitted in the Nurses Home. I guess somebody felt that protection from males was needed for the nurses who at that time were all young ladies! I went back to the hospital where I had been told House Staff were lodged. Indeed I found a room but only for one week. After that I managed to find a room, again in the Hospital but this time under the Dome. These were much larger rooms with windows looking right on Broadway. I was told that they were usually reserved for Chief Residents. I wonder whether present administrators who occupy these offices now know what went on in the early days in this space. After hours, the residents would meet, have great discussions, sing songs with one of them playing a guitar. Right under the Dome there was also a ping-pong table where many a disputed game was played. It was also a fact that it was not quite as sex-segregated as the Nurses’ Home.

When I reported this observation to Dr. Wilkins, he stated that the house-staff members could do what they wanted when off duty. He added that, at the time of “Prohibition” when he was a medical student, ingenious colleagues had learned how to ferment various mixtures in the bathtubs of their rooms and to distill the brew into a strong but ugly alcohol. With time, the students had refined their technique: adding orange juice and sugar to the distillate which resulted in something resembling Grand Marnier. And we both laughed. What amazed me was I could comfortably discuss such escapades with my new Chief and I enjoyed it.

My room under the Dome was also a temporary lodging. Next, I moved to a fraternity house that was located on Broadway, across from the hospital. This too was very temporary. These fraternity houses were replaced a few years later by a church and a Holiday Inn, which were replaced a few years later by the present Outpatient building.

Eventually, I learned about another French fellow, Dr. Jacques Monier of Toulouse who was working with Drs. Eleanor Bliss and Caroline Chandler studying new antibiotics in the department of Dr. Perrin H. Long at the School of Hygiene. Dr. Wilkins explained to me that Dr. Long was a pioneer in the study of chemotherapy starting with sulfonamides.

I told him that I had been impressed with this therapy: the American soldiers in France seemed to all have a plastic box with sulfa-powder that they used on any of their wounds with great results.

Jacques Monier had come with his wife, Evelyn, a nurse who was working at the Hopkins Nursing School. The three of us decided to rent a small row-house on Madeira Street, near the Monument Street Food Market. I had discussed that option with Dr. Wilkins who thought that it was a good option: it would give me some relief from the English language! He laughed when I told him that every evening, Evelyn and Jacques prepared a large glass of water with a bunch of aspirin tablets, each of us being given a part of the glass in proportion to the hardship of our day.

For distraction, we bought a second-hand record player, but very few discs. I told Dr. Wilkins that now I knew by heart the violin concerto of Beethoven as played by the Philadelphia Orchestra directed by Eugene Ormandy with Zino Francescatti playing the violin. Dr. Wilkins confessed that he had received violin lessons in his youth but that he was not very fond of high-brow music; he preferred Gilbert and Sullivan’s music and he proceeded to give me a few bars of the Pirates of Penzance. He always had a way to be warm and gracious.

After one year, the Moniers went back to Toulouse, but I stayed on. Dr. Wilkins had obtained support for me from the André and Bella Meyer Foundation for a second year of fellowship. I moved to a third floor apartment above the Fish laundry at the corner of Broadway and Monument Street: very close to Hopkins but with lots of rats in the backyard at night. I shared this apartment with Gordon Kennedy, a physician from Cambridge, England who had come to work in our Pediatric Endocrine Clinic. I don’t know where Lawson and Gordon had met. Gordon was an expert studying brain lesions in rats which resulted in polyphagia and obesity. Wilkins was impressed by it, and interested in that work.

Gordon was an outspoken man who expressed himself in a forward fashion. One day, Gordon and I met Wilkins, who appeared overjoyed in announcing that Mr. Winston Churchill had been elected. Gordon–a very liberal person–answered: “That big sh–.” Personally, I was upset by his remark and I left the room, trying to avoid further conversation about it. This difference of opinion did not affect our friendship: A few years later the Wilkins’s went to visit the Kennedys in Cambridge and later, I went to see Gordon and his wife in their home: a fun experience with a lot of pints at the pubs! As to Mr. Churchill, his premiership was quite short and he retired from the political arena.

## In my new office with Dr. John Crigler

**Figure 6:**
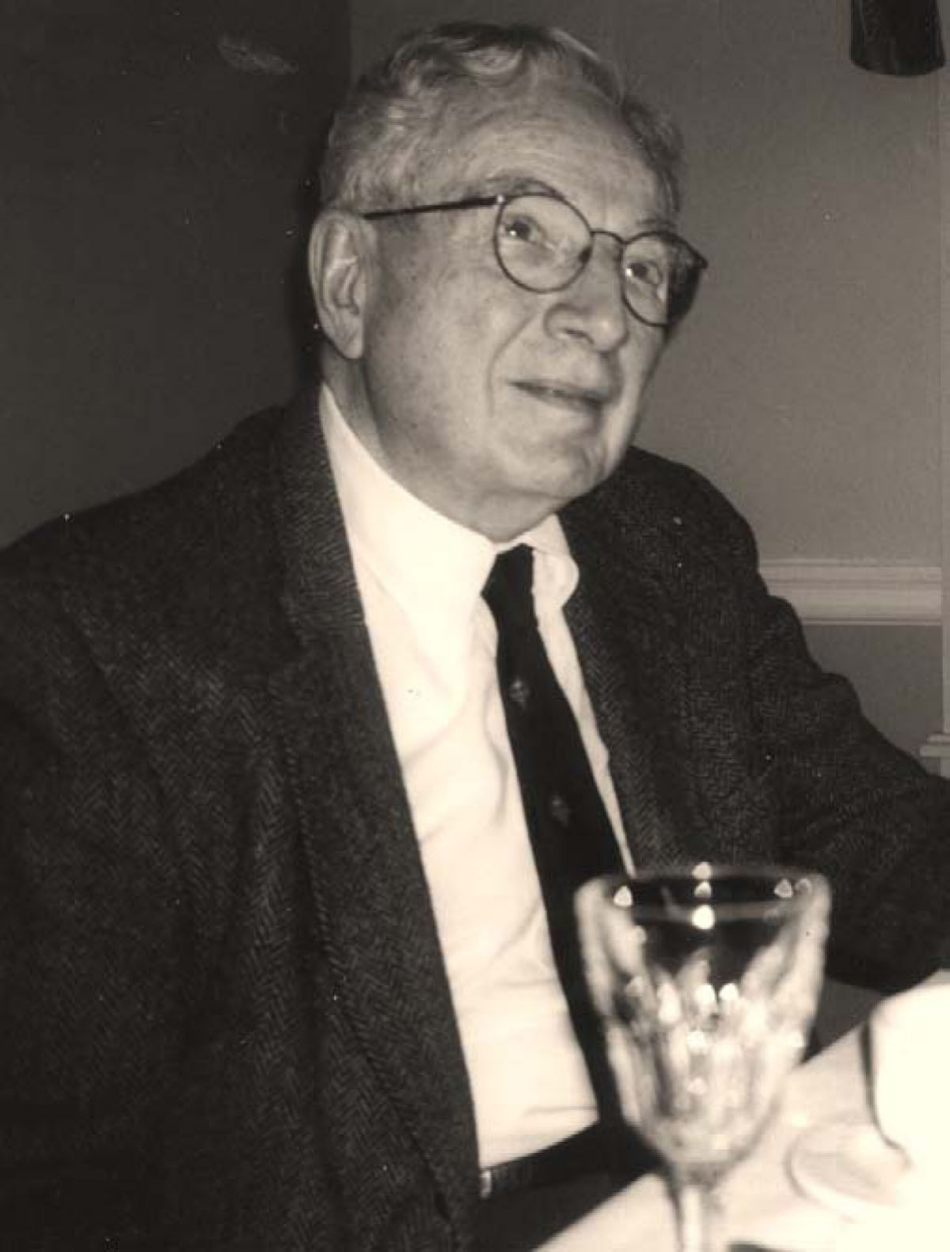
John Crigler, one of the early fellows. (April 2000)

Sharing an office with Dr. John Crigler (Figure 6) was an important way to get information on the Pediatric Endocrine Clinic and its chief Dr. Lawson Wilkins.

John was very loquacious. So first, I learned that he had entered Johns Hopkins Medical School in 1939, the year he got married to his lovely wife, Marie Adele, who had a marvelous Southern accent from North Carolina. He had started an internship in Medicine but shortly thereafter he joined the US Navy in World War II. This statement raised my interest as I had been involved in the years of WWII. On D-Day, he was on one of the boats (probably in the second wave) as a physician. He stayed on his boat, under plane attacks, tending to wounded men.

I explained that, during the war, I volunteered in a small hospital in Rethel (Ardennes). The physician in charge decided to put the patients in the basement to avoid the bombs. I helped to transport them to the cave by passing through a huge open courtyard. When we were halfway there, we encountered machine gun fire from a plane passing over us but they missed. At that point, we ran for shelter.

John and I both enjoyed sharing our adventures, including the hits and misses. And we certainly talked about much more.

The following is what John Crigler wrote as a historical summary of the highlights of the Wilkins era at Johns Hopkins:

## Years 1935-1946

Wilkins organized the first subspecialty Clinic of Pediatric Endocrinology in the world. He initiated longitudinal clinical observations, especially of physical growth and development of infants, children, and adolescents with growth disorders and endocrinopathies. He also established a biological laboratory under the direction of Dr. Walter Fleischmann, a refugee from Nazi Germany who was a distinguished biochemist. He carried out hormonal and metabolic studies in animals and human beings.

His principal research interests were the following: thyroid disorders and the effect of thyroid hormone on skeletal growth and epiphyseal development and on creatine/creatinine and cholesterol metabolism; beginning of studies on abnormalities and variations of sexual development during childhood and adolescence including the publication with Drs. Fleischmann and John Eager Howard of papers describing “Macrogenitosomia precox associated with hyperplasia of the androgenic tissue of the adrenal and death from corticoadrenal insufficiency” in 1940, [1] “The metabolic and growth effects of various androgens in sexually immature dwarfs” in 1942, [2] and, without Howard, “Ovarian agenesis: pathology, associated clinical symptoms and the bearing on the theories of sex differentiation” in 1944 [3] and “The influence of various androgenic steroids on nitrogen balance and growth” in 1946 [4].

It is important to note that all of this was accomplished while Dr. Wilkins carried on a large fulltime practice and without compensation from Hopkins. Evident during this time was his broad interest and involvement in clinical endocrinology (minus diabetes mellitus) as he established the program with an investigative focus on the metabolic effects of thyroid and steroid hormones. Dr. Wilkins’s ability to work with associates in other departments such as Dr. Howard in Medicine and Dr. Young in Urology significantly enhanced the development of his program. It also was during this time that he attended a periodic (I believe it was at least yearly) get together of a very select group of endocrinologists (from basic science – Dobriner, Pincus, etc. and internists – Albright, etc.) who essentially became his mentors and he their mentor in human developmental endocrinology. I suspect the group had a name but I don’t remember it at this time if it ever had one. Dr. Albright was the individual in this group who had the biggest impact on Dr. Wilkins.

## Years 1946-1950

Wilkins became fulltime at Hopkins; he established a postgraduate training program as we recognize it today. He published the first textbook of **Pediatric Endocrinology** (1950), importantly based on his meticulously documented and systematically organized and recorded longitudinal clinical observations related to pathophysiologic mechanisms. He reported the unsuccessful treatment of an infant with salt-losing congenital adrenocortical hyperplasia (CAH) with total adrenalectomy [5] and the successful suppression of androgen began our first experience with using cortisone in such patients. For me, it was a Baltimore summer spent maintaining the well-being of salt-losers under study and working with a flame photometer in a closed room (without air conditioning), which was the beginning of the long-term studies on salt-losing CAH carried out during my year as a fellow and subsequently reported. [6] What remarkable luck to come along at such a time and to be with such great colleagues.

We had weekly conferences with the endocrine group from the Department of Medicine under Dr. Howard, weekly Saturday clinics, and continuing studies on the effect of various glucocorticoids on the hormonal and metabolic responses of patients with different manifestations of CAH which resulted in a series of papers in Journal of Clinical Endocrinology and Metabolism in 1951-1952 and defined some of the pharmacological requirements for longer term treatment via different compounds and routes, dose and schedule of administration.

## Establishing the treatment of congenital adrenal hyperplasia (CAH)

For the readers who are not endocrinologists, it might be helpful to outline our present knowledge of CAH. It is an inborn error of metabolism caused by a mutation of one of the genes that encodes the enzymes needed for cortisol and aldosterone synthesis from cholesterol. As it is an autosomal recessive disorder, both parents must contribute a mutant gene to the affected child. More than 90% of CAH cases are due to a deficiency of 21-hydroxylase, an enzyme the gene for which is located on chromosome 6, in the middle of the very important HLA genes: HLA A, B, C, DR, DP, and DQ.

In major 21-hydroxylase deficiency, both cortisol and aldosterone secretion are deficient, resulting in the *salt-losing form of CAH*. In milder deficiency, only cortisol synthesis is affected: the *simple virilizing form* of the syndrome. In both forms, the output of adrenal androgens is excessive because of over-secretion of ACTH and accumulation of precursors of cortisol which generate androgen synthesis. This will cause masculinization of the external genitalia of female fetuses including enlarged clitoris and variable degrees of fusion of the labia.

This knowledge was not available in 1950 when I arrived at Harriet Lane. John Crigler gave me a succinct review of what was already known about CAH and its treatment.

Three or four years earlier, Lawson Wilkins and Walter Fleischman along with Roger Lewis and Robert Klein had attempted to treat these patients with various synthetic steroids in the hope of suppressing the excessive production of androgens by their adrenal glands. Although these studies had generated interesting data on the anabolic effects of those various steroids, they failed to suppress the production of androgens in CAH. Fuller Albright at the Mass General Hospital in Boston had proposed that the adrenal cortex of human subjects was secreting three major groups of hormones: one he called the “sugar hormone,” another was the “salt-retaining hormone,” the third was the “adrenal androgens.” On the basis of this model it was easy to visualize that the abnormality in CAH was overproduction of the male hormone with a decreased secretion of the sugar and salt-retaining hormones. Roger Lewis, Robert Klein and Lawson Wilkins had shown that the administration of ACTH to CAH patients produced a further increase in adrenal androgens measured as urinary 17-ketosteroids. At the same time they observed that ACTH administration caused a greater disturbance in the salt balance suggesting that perhaps ACTH was increasing the secretion of a salt-losing hormone.

With a great deal of pride, John Crigler explained to me that many major endocrinologists at Hopkins had been involved in the preparation of adrenal extracts which could help the salt-loss of patients with Addison disease. And he cited the names of George Harrop, George Thorn, Harry Eisenberg, Lewis Engel, and John Eager Howard. In the late 1930's, Reichstein and colleagues in Basel synthesized deoxycorticosterone acetate (DOCA). This steroid did help the salt imbalance but did not correct the hypoglycemia.

Dr. Wilkins added to this conversation, stating that the sugar hormone, 11-dehydro-17-hydroxy-corticosterone, now known as cortisone, had been isolated from extracts of the adrenal cortex by Dr. Kendall at the Mayo Clinic in the mid-1940s. A sample of this preparation had been given to Dr. George Thorn who showed that it was effective in correcting the hypoglycemia of patients with Addison disease (adrenal insufficiency). It took several more years for the Merck Company to synthesize cortisone acetate in amounts large enough for therapeutic trials.

In early 1949, cortisone acetate was used to treat patients with chronic arthritis. I remember a movie that showed patients unable to ambulate before treatment. After two weeks of cortisone acetate, they were able to walk and step up on a ladder. This was the first use of the new hormone.

**Figure 7:**
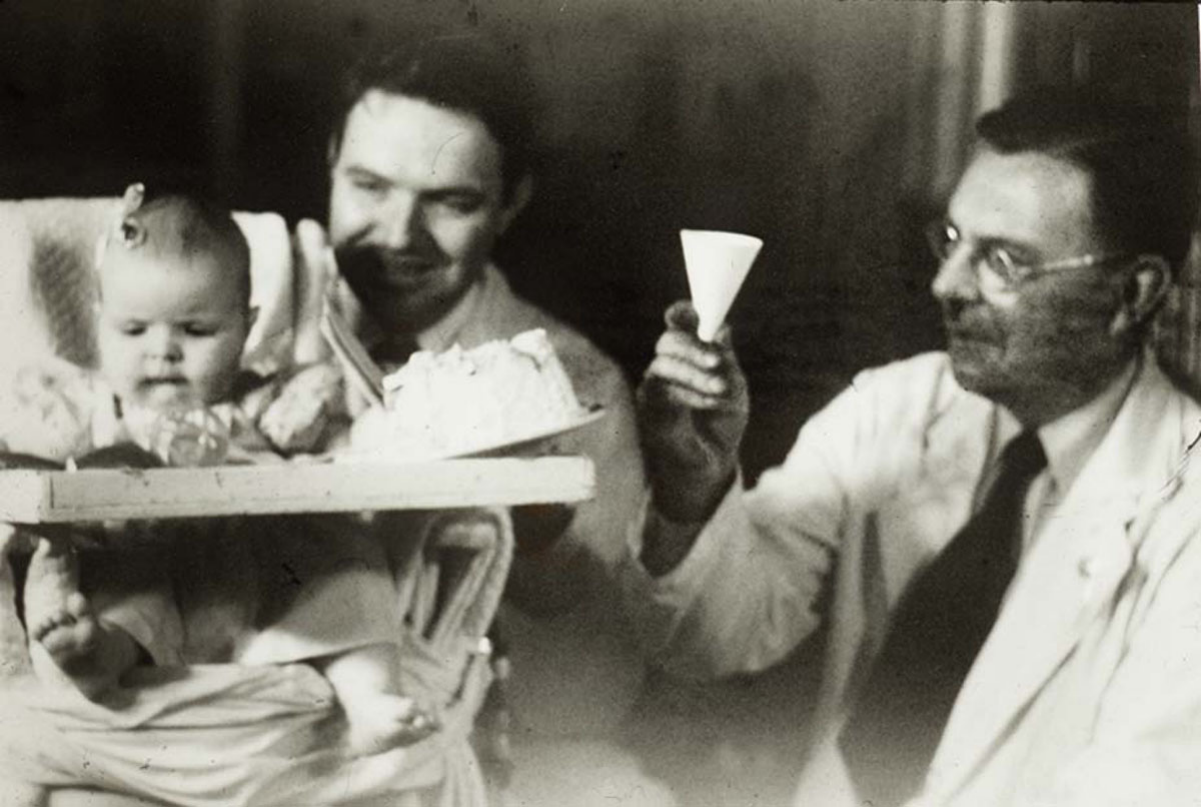
Loys at her first birthday with Lytt Gardner and Lawson Wilkins. Loys was the first baby with the salt-losing form of congenital adrenal hyperplasia who survived with treatment of cortisone, salt retaining hormone. (1951)

In late 1949, Dr. Wilkins, along with Drs. Roger Lewis, Robert Klein and Eugenia Rosenberg, studied a fifteen year-old girl with CAH. When she was six years old, part of her adrenal glands had been removed by Dr. Hugh H. Young but this did not influence the excessive adrenal androgen secretion. However, the administration of 100 mg of cortisone acetate daily for fifteen days dramatically suppressed her urinary excretion of androgens (17-ketosteroids). Simultaneously, Fred Bartter and the endocrine group at the Massachusetts General Hospital in Boston obtained similar results. This was a great achievement in the treatment of abnormal adrenal function. The first patient with salt-loss who survived is shown on her first birthday in Figure 7.

Further investigation by Wilkins and his fellows, as well as other researchers illuminated the pathophysiology of CAH. We learned that the adrenal glands of these patients are unable to produce the “sugar hormone” (glucocorticoid, cortisol). In an effort to compensate for this, the pituitary gland secretes large amounts of ACTH resulting in excessive production of adrenal androgens. Treatment with cortisone replaces the missing sugar hormone and at the same time suppresses ACTH and androgen secretion.

From 1952 to 1955, Alfred Bongiovanni and Walter Eberlein were at Hopkins, studying the steroid metabolism in CAH patients. They concluded that there was a block in the steroid biosynthesis at the level of the 21-hydroxylase. It resulted in insufficient secretion of cortisol with an increase in precursors such as 17-hydroxy-progesterone which were metabolized to the virilizing androgens.

## Learning how to measure the urinary excretion of steroid metabolism

After my short indoctrination on the treatment of CAH, I asked Dr. Wilkins how I could help. At that time, we knew that cortisone acetate treatment could suppress the adrenal androgens. However, very quickly it became evident that it was important to determine the minimal amount of cortisone needed to suppress the adrenal cortex as excessive amounts resulted in signs of Cushing syndrome. The only means available to determine the degree of androgen suppression was the measurement of the 24-hour urinary excretion of 17-ketosteroids (17-KS). Hence, patients were admitted to the hospital, and given various amounts of cortisone. Urine collection was a key step in the study and the measurement of 17-KS was a tangible end-point.

There were many problems in measuring the urinary excretion of 17-KS. It was most important that the 24-hour urine collection be complete. This was difficult enough in toilet-trained children, but it was a major problem in infants. The Harriet Lane nurses devised ingenious means for urine collections in both boys and girls. Another problem was the storage of the urine samples. The small cold-room was overflowing with urine bottles. Freezers had to be purchased as it seemed important to store some samples for further investigations at a later date.

The laboratory technicians, Mary Ellen Crafton and Henry Schulte, and shortly thereafter Betty Youngblood-Lawrence, were expected to run all the tests. However, the volume of work was overwhelming. So I was most welcome to learn how to measure the “ketos:” boiling an aliquot of acidified urine, extracting steroids with ether using separatory funnels, washing the extract with sodium hydroxide and water, carefully evaporating the ether and then, carrying out a Zimmermann color reaction on the extract. All together, we did thousands of Zimmermann reactions and “ketos” measurements.

One day, out of nowhere, appeared a middle-aged gentleman who introduced himself, “I am Dr. Zimmermann, with two “ns” at the end, of the Zimmermann reaction.” I was quite surprised as I expected the man to be part of past history. When I presented Dr. Zimmermann to Lawson, I believe that he was also surprised but he did not forget to ask him to sign his visitors’ book. Later, I realized that, in our publications, we had used “only one “n” at the end of his name!

The laboratory performed several tests in addition to the 17-KS assay. There was the bioassay for gonadotropins using immature mice. We measured urinary 11-oxosteroids and total estrogens. This latter was measured as “estroids” in urine.

Because urinary “estroids” were elevated in untreated CAH and decreased with cortisone treatment, I suggested to Dr. Wilkins that further studies were needed. He agreed that it was an important question. In order to carry out estrogen bioassays, he helped me contact Dr. W.W. Scott in Urology. Then, he arranged for me to visit Dr. Lewis Engle’s lab in Boston and paid my travel expenses. This is how I got involved in the separation of the various estrogens–estrone, estradiol, and estriol by counter-current distribution. When I wrote the results for publication, I asked Dr. Wilkins to review and correct my text. The next day, he returned my paper with no corrections, along with a new edition of this paper, fully hand-written by him. He explained that it was easier for him to re-write rather than correct. I had the hand-written version typed by the secretary and made sure that Lawson Wilkins was an author. I re-submitted the paper to him and it came back to me with two corrections: Lawson’s name was deleted from the front page and an acknowledgment to Dr. Wilkins was added at the end.

On that day, I realized that under the cover of roughness suggested by the gruff voice, there was a huge amount of generosity and warmth in this man. On that day he became more than my mentor: he was more like a father.

## Establishing the details of treatment of patients with CAH

In the evening of each working day, the ketos results were posted on each patient file. The files were pinned on bulletin boards in the secretaries’ office. After the secretaries had left, Dr. Wilkins was joined by Lytt Gardner, John Crigler and me. Often, there would be one or two interns as well as one of the numerous visitors. Long discussions would take place about modifying the treatment of each patient. Some studies compared results obtained with oral versus intramuscular cortisone–or compared cortisone to cortisol, or to corticosterone. These sessions were fascinating. Anyone could give an opinion but the discussion was usually dominated by the deep bass voice of Dr. Wilkins and the high-pitched voice of John Crigler.

In the early ‘50s, it seemed that everybody smoked. Our little meeting room would rapidly fill with the smoke of burning cigarettes. Dr. Wilkins was the worst; each day he would come with two packs but often it was not enough; we knew to have cigarettes available for him at the end of the day in order to fill the gap. We joked that Dr. Wilkins used only one match per day, the one that lit his first cigarette.

**Figure 8:**
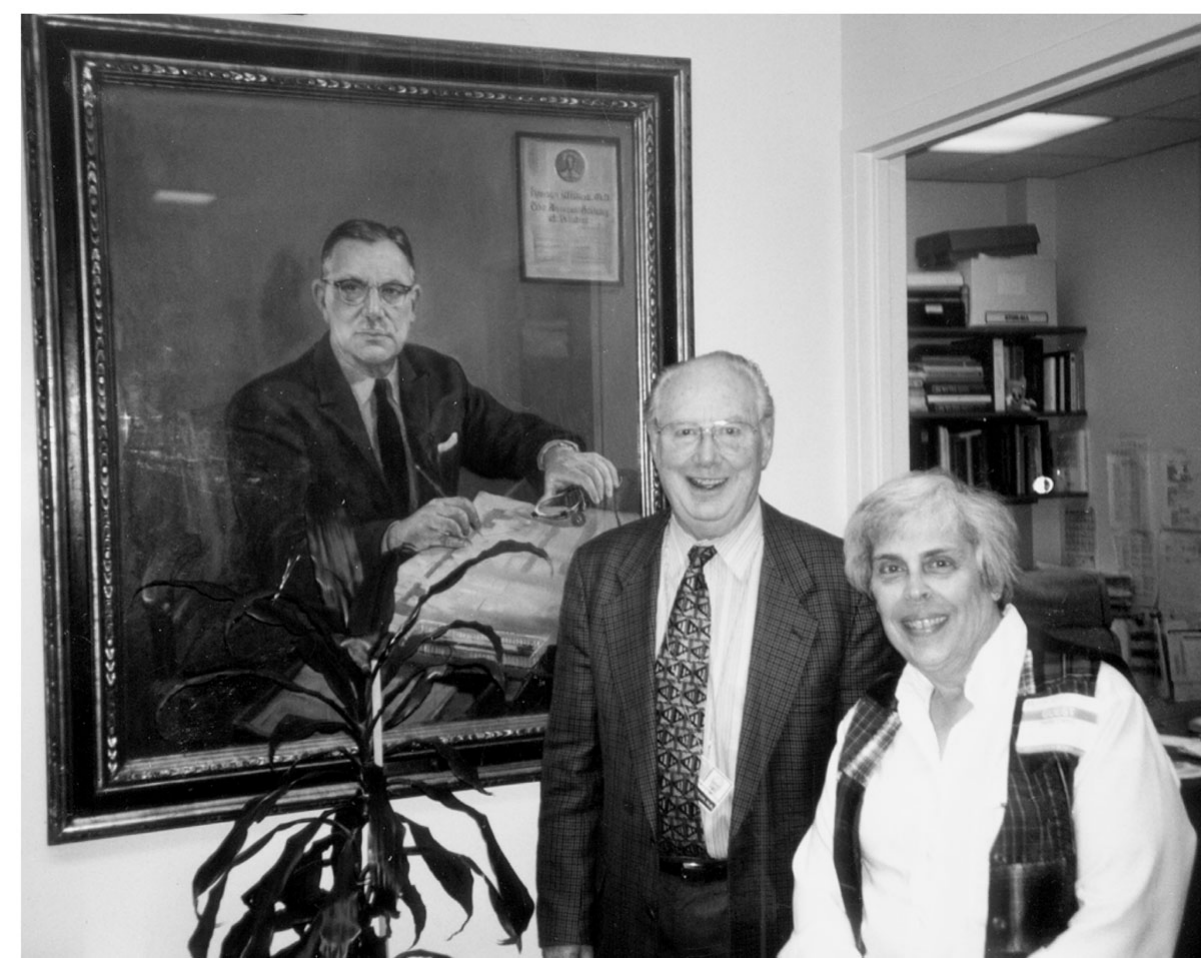
Claude Migeon with one of the very first non-salt-losing patients. (1990)

It seems that out of these daily meetings came the elaboration of the proper treatment for CAH patients. We learned that oral cortisone acetate had to be given three times a day, whereas IM cortisone acetate could be given once every three days. Further, the IM preparation was twice as potent as the oral tablets. An additional observation was that some patients presented in acute adrenal crisis with salt-loss in the first 5 to 7 days of life (salt-losing form) whereas others presented with masculinization and hirsutism only (simple virilizing form). (Figure 8)

## The psychohormonal research unit

Shortly after my arrival at Hopkins, Dr. Wilkins mentioned to me that he had met in Boston a young man from New Zealand who was finishing his Ph.D. at Harvard. The subject of his thesis was the study of patients with abnormalities of sex differentiation including patients with congenital adrenal hyperplasia. His name was John W. Money. Wilkins thought that this man would be of great help with the many patients we were seeing in our clinic who had various problems of sex differentiation.

I cannot do better than to quote Dr. Wilkins in the preface of the Second Edition of his textbook:

“We have been most fortunate to have working with our group of pediatric endocrinologists a team in Psychology and Psychiatry, John W. Money, Ph.D., Joan L. Hampson, M.D. and John G. Hampson, M.D., under a special grant from the Josiah Macy, Jr. Foundation. Their studies and management of the psychological problems of adjustment which confront the patients with various types of endocrine disorders have been of the greatest benefit to us and to the patients. In addition, the endocrinopathies produced deviation from the normal path of somatic and sexual development which presents an extraordinary opportunity to evaluate the relative roles of environmental, hormonal, and chromosomal factors in determining psychology patterns. Some of their findings have had a profound influence on fundamental concepts of psychosexual development.”

Indeed John Money and Joan and John Hampson were extremely helpful in the management of our pediatric endocrine patients. John Money described it well in the preface of the Third Edition of the Lawson Wilkins textbook:

“There was another sequel of the adrenogenital story for which Wilkins has not, until now, received the proper amount of recognition; namely the establishment of a new psychohormonal research unit for the study of psychology of hermaphroditism and related endocrine disorders. This psychologic research has increased fundamental knowledge of the process of psychosexual differentiation relative to genetic and hormonal variables and the sex of rearing.

It is a sign of Wilkins’ breadth of intellect that he surmounted a traditional bond of prejudice and sponsored psychologic investigation in his clinic...”

The work of the Psychohormonal Research Unit raised a great deal of controversy. It was suggested that Money was ignoring “nature” and overemphasizing “nurture” in the nature-nurture conflict in matters of psychosexuality. In fact, the Research Unit has, all along recognized the dual effects of both nature and nurture.

## Dr. Wilkins’ Saturday morning clinic

Dr. Wilkins saw patients almost every morning. This included Saturday. He would see a limited number of patients–three or four–at each clinic. Each day one of the fellows would help him with the clinic in “Observation 3" (down in the lower floor of the Harriet Lane Home).

Every clinic of the week was interesting and educational. However, the Saturday Morning Clinic was the jewel of them all. Dr. Wilkins would specifically schedule patients with unusual conditions or with a long follow-up that would permit a critical evaluation of the therapeutic results.

The entire staff of the Pediatric Endocrine Clinic was expected to attend. On occasion, I would attempt to finish an experiment in my lab. Chances of escaping from Saturday clinic were nil: Dr. Wilkins would make a run to the 5^th^ Floor, asking Odessa to hold the elevator for his return downstairs. Quickly, he would come to tell me that there were very important cases to be discussed. And, of course, I would rush down with him to the conference room.

The large conference room with two sofas in a circle was available for discussion. Each patient would first be seen by one of us in an observation room, and then the history would be presented with great detail in the conference room. Dr. Wilkins would ask advice of the attendees who might have special knowledge of the problem and discussion would ensue, new ideas would be debated.

In addition to our Staff, there were usually two or three interns and residents. There would also be visitors from the US or abroad. Dr. Marvin Cornblath, a pediatrician serving in the Army at Fort Dietrich in Frederick, Maryland would attend every week. John Money and his assistants of the Psychohormonal group were part of the group. Dr. Wilkins would also invite members of Pathology or the Medicine faculty to discuss unusual disorders.

Every clinic was a great success and by 1:30 p.m. we would all rush to the “Doctors Dining Room” which closed at 2:00 p.m. We would sit at a large round table where the discussion of the cases would continue. Every time Dr. Wilkins would conclude: “That was a good clinic” partially as self-satisfaction but also justified by the interesting comments of the various experts.

Sometimes, Dr. Helen Taussig would join us for lunch. She was very pleasant. It was said that when she did not like the discussions, she would turn off her hearing aid system.

## Experimental work and language difficulties

The Pediatric Endocrine Clinic under Dr. Wilkins provided a stimulating environment and an atmosphere of enthusiasm for research. At our Journal Club, we all contributed to the discussion of recent publications. One novel idea, brought to my attention, was the effect of DDT (dichlorodiphenyltrichloroethane) on humans and particularly on the adrenal gland. Dr. Wilkins and Dr. Gardner suggested to me that experimental work on dogs and might be useful in clearing up this problem.

Arrangements were made through Dr. Stanley Eversol, the Chief Resident in Pathology to provide six dogs who would be caged on the top floor of the Pathology building. After much discussion with Dr. Wilkins, we agreed on a specific protocol. Every day I would go and visit the dogs, to check on the amount of DDT which was fed to them with their regular diet. I was told that the dogs were well taken care of and exercised every day. We carried out the Thorn test of adrenal function, which involved an eosinophil count before and four hours after an injection of ACTH, twice a week.

Then one day I found that two of the experimental dogs had baby dogs in their cages. I was also told the third one, a female, was also pregnant.

It became clear that the exercise that the dogs were taking was communal. It was also clear that treatment with DDT did not decrease the interest of the male dogs for their female partners. That day when I came back from the Pathology building to Harriet Lane, I was very distraught. When Dr. Wilkins asked me how the experiment was going, I told him that the dogs had “poopies.” Lytt Gardner, who was close to us, added, “Claude means puppies.” Well, Dr. Wilkins had a good laugh, and at that point I realized that not only my experimental work was a fiasco but that also I had created folklore for the future. In fact when my name came up in a conversation, Dr. Wilkins would say “Claude doesn’t work anymore with dogs because they have poopies.”

Dr. Wilkins loved to have fun and in the process, I learned how to pronounce certain words properly.

On another occasion when I had visited Boston I had stayed overnight with friends of Lytt Gardner. On my return, I had asked Dr. Wilkins how I should express my thanks. He suggested I could send a little note to the hostess. I did so and later presented my draft to him. At that point he said: “Claude, I think you might want to change the sentence which goes ‘Thank you very much; I very much enjoyed the night I spent with you.’ I would replace the word ‘night’ with ‘evening’.” I realized what I had written and we both smiled. The fifth floor of Harriet Lane, specifically the Pediatric Endocrine group, was the site of a lot of hard work, a lot of scientific discussions, and also a lot of fun.

## Riding with Dr. Wilkins

On a first encounter with Lawson Wilkins, most would have considered him quite somber. Most of the time, he would appear very serious. He usually wore a charcoal suit with a white or blue shirt with a button-down collar and a dark tie. Once, he took me to S. Schwartz, a large discount retailer, as we both needed a new suit. I settled on a light brown. Dr. Wilkins bought another charcoal suit.

This sullen appearance was a cover-up for a very sociable and congenial personality. Dr. Wilkins loved to talk. Indeed he had a deep need to communicate, to share his own thoughts.

Often he was invited to give lectures: at the NIH and the Naval Hospital in Bethesda and at the Union Memorial Hospital in Baltimore. Sometimes he would give a series of lectures covering the major aspects of Pediatric Endocrinology. Almost routinely, he would ask if I wanted to come along, suggesting that I might be interested in the subject of the lectures or that I might help in showing the slides. Each time, it was an occasion for a trip in his car. And of course, it was an opportunity for conversation. In my early days in Baltimore, my English was poorly enunciated and not easy to understand.

So, Dr. Wilkins would carry out the major part of the discussion. We considered many subjects, ranging from aspects of the work in the clinic, to the personality of people at Hopkins and outside of Hopkins.

One day, driving on Broadway from Fells Point to Hopkins Hospital, we stopped in front of 6 North Broadway. There was an elegant brick house with a stone base where his father practiced medicine and where Lawson was raised as a child. The side of the house was on a small alley. Dr. Wilkins showed me the garage where the horse and buggy had been kept. He had great memories of the horse and buggy used by his dad to visit his patients. He remembered the first motorcars which had trouble going up the hill of South Broadway. Sometimes, with other kids, he would give the weak cars a helpful push up the hill. And he remembered what great fun it was.

This was also the house where, at the time of the great Baltimore fire, he was kept on his knees with his sister in order to pray for the safety of the city when he would rather have watched this great fire, and the firemen fighting it.

## Skip’s accident (January 15, 1944)

Another time, driving on Cold Spring and arriving at Dolfield Avenue, Dr. Wilkins told me that this was where his 16-year-old son had been killed. In the winter of 1943-1944, Skip had a Christmas job driving a post office truck. Another car ran into him and he was ejected out of his seat. Dr. Wilkins would further describe how he had to identify his dead son at the morgue. One could almost palpate the depth of his sorrow. Saying that I was sorry did not come close to match the sadness of Lawson. And a silence would take over for a while.

**Figure 9:**
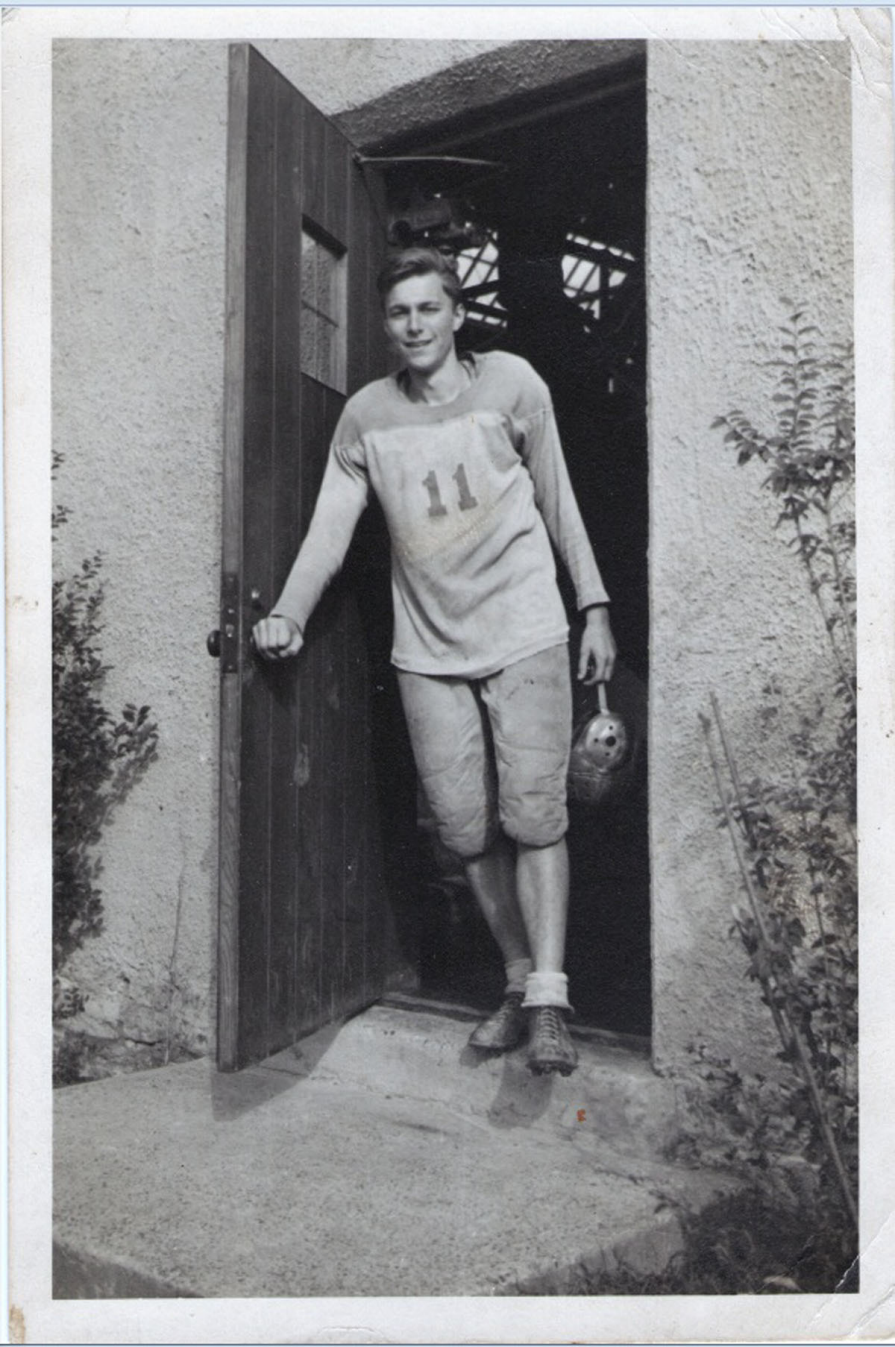
George Lawson Wilkins II—“Skippy”. 1927-1943

The subject of his son would come up quite often, usually in late evening, after he had a few glasses of rye whiskey. “I guess Skip was not a scholar, but he was a kind boy” or “I don’t think Skip would have made it in medical school.” Then the tone of his voice would go deeper and its volume would go down, before a short silence. Then, the conversation would resume on another subject. (Figure 9)

There was an incredible mournful sadness in Dr. Wilkins’ soul when it came to the loss of his son. But I would give a totally wrong impression if I had suggested that he was a sad person. On occasion, after a hot day on the 5^th^ floor of Harriet Lane, he would bring me to his house on Edgevale Road. In summer, when we would hit University Parkway, he would note that the temperature had gone down at least 5 degrees and that was good! And since it was still daylight, we could have a game of croquet in the backyard with Mrs. Wilkins. These sessions were riotously funny.

## A Christmas dinner in Baltimore (December 1950)

Both Dr. Wilkins and Lucile had a great interest in French cooking. They asked me to describe a typical French Christmas dinner. I explained that, in my family, this was a very elaborate meal, lasting from noon to late in the evening, usually including soup, fish, turkey, vegetables, dessert with wines, champagne and coffee. Dr. Wilkins became excited and wanted to experience such a dinner. He proposed that Lucile, Mary Adele Crigler and Lockley Gardner could fetch all the ingredients needed. All I would have to do is make the appropriate list of supplies and organize the ladies, who would do the work in the kitchen and the dining room. How I let them convince me that it would be easy I shall never know. The site for the now famous dinner was the row house on Wolfe Street where the Criglers lived. The date was chosen as a Sunday night before Christmas, as everyone had previous commitments for December 25.

Several weeks ahead of the date, I communicated with my aunt who sent me a recipe for the “Buche de Nöel”, the traditional Christmas cake, shaped like a log, which was very difficult to make! Every day, Dr. Wilkins wanted reassurance that I had not forgotten anything on my list, requesting supplies, particularly the wines. Mrs. Wilkins, Mrs. Gardner and Mrs. Crigler were busy finding the essential ingredients. Buying a turkey was easy, but finding the truffles to slip under the skin was much more difficult. Dr. Wilkins told me that the ladies had to take a trip to Washington, DC to purchase a little can of truffles, and that it was amazingly expensive!

As the day of the festivity came closer, the excitement grew. On several days, I went to rehearse my helpers. Who was going to prepare the home-made mayonnaise ahead of time? This required Dijon mustard (real!), peanut oil, and very fresh eggs. When would the green beans and potatoes be prepared? Dr. Wilkins found out that “colin,” the fish that I needed, was “hake” in English. From his home, he brought the proper container for poaching the fish, and a long plate to lay the “colin” for serving. Mrs. Wilkins obtained the chestnuts for the stuffing of the turkey. Mrs. Gardner purchased the French unsalted butter and the pistachio for the Buche de Noel.

Early Sunday morning, I came to the Crigler’s home. I started with the “Buche de Nöel”, cooking the sponge cake and preparing the chocolate-butter cream. This could be done in advance and kept in the icebox. The same for the “colin.” I had a problem with it: when I tried to remove the skin and bones; it all fell apart. But, with my hands I rebuilt a fish shape on a bed of leaves of lettuce on the serving plate, and then covered the irregularities with mayonnaise that I made myself. For the turkey, I made small cuts in the skin and introduced a thin slice of truffle between skin and meat. My lady-helpers took care of the chestnuts: we added a very small amount of bread stuffing and, most importantly, a small glass of cognac. The helpers also took charge of the cooking. Then, for the soup, I used beef bouillon, adding onions sautéed in butter. Before serving it, we added bread croutons and grated Swiss cheese. Preparing the green beans and potatoes was fairly simple. They were spiced with garlic, parsley, and lots of butter.

A great discussion had taken place the previous day about an apértif before the meal. Lytt Gardner, a well-recognized expert in mint juleps had offered his services. Lawson preferred Scotch. I had proposed the French sweet vermouth, Cinzano. Eventually, we agreed that as they would spoil the meal, pre-dinner drinks could be dispensed with. So the memorable menu is in additional file 1

This was quite a feast! The Grand Marnier liquor brought up the story of Lawson making some orange liquor when he and his colleagues were medical students. This was done with oranges, their peels and of course, alcohol. The maceration took place in the interns’ bath tubs at Hopkins, hidden from the authorities. This, in turn brought up the story of the Hopkins Unit in France during WWI. An officer called Lawson and his colleague student-soldiers to attention. Their names were read and it was announced that they had received their MD degree. This was their graduation ceremony. After that, they were told to go back to their occupation: on that day, it was latrine duty!

Needless to say that in the following days, Lawson Wilkins reported to whoever was willing to listen the story of this memorable dinner. He would describe with great detail how I handled each individual crouton, in order to make sure that they were all grilled just right on every side. He also told how he had to carve a turkey, which had truffle bruises all over its skin! “But it was magnifique!”

## Celebrating the New Year in Chicago with friends (January 1951)

Dr. Wilkins had invited me to join his family for the New Year. He said that I was not asked to help with the cooking, like I did for Christmas. He noted that the dinner might not be as good but it would be great fun.

I had to explain that I was going to Chicago to visit my friends, Bill and Stella Nanos. I explained that I had met them in Paris, that we had created a great amity and love. Together, we had visited the grave of their brother who had died while he was with the American troops at Bastogne, Belgium on Christmas Day of 1944. Of course, he understood and made me promise that I would visit him when I got back.

Indeed, I did tell Lawson about my Chicago trip. Not all at once, but on several occasions while we were driving together. One time, I explained that my friends, the Nanos, were of Greek origin. Stella had a Master’s degree in Biology and she trained with Dr. Papanicolaou, the man who established the “Pap smear.” Stella directed the cytology lab at the hospital of the University of Illinois. Bill was involved with buying books for the students at the University of Illinois.

It was so nice to be together again and my friends had a marvelous surprise for me: they had tickets for the show *South Pacific* with the original cast: Mary Martin and Ezio Pinza. The musical is based on the book “Tales of the South Pacific” by James Michener. The 1949 Broadway Production had music by Richard Rodgers and text by Oscar Hammerstein.

One of the songs, sung by a little girl, is in French:

“Dites moi pourquoi la vie est belle

Dites moi pourquoi la vie est gai

Est-ce que c’est parce que vous m’aimez?”

Martin was wonderful when she sang “I’m Gonna Wash That Man Right Outta My Hair” while Pinza was marvelous singing “Some Enchanted Evening.” I told Wilkins that the show was terrific and that he should see it, which he did later in New York City. He loved it as much as I did.

The week with my friends was quite short. With some of their friends, we went to the best French restaurant in Chicago, visited the art museum, whose numerous paintings by French Impressionists amazed me.

I do have to say that the weather was quite rough with snow and wind coming from Lake Michigan. I had no problem with my flight back to Baltimore, except for the sadness to say goodbye to Bill and Stella Nanos.

## Presentation of the results of the treatment of congenital adrenal hyperplasia with salt-loss: Atlantic City (June 1951)

In 1950 and 1951, all of us – especially John Crigler – had focused our attention on the treatment of infants with the salt-losing form of congenital adrenal hyperplasia. First, we found that these infants needed cortisone as well as the salt-retaining hormone. At that time, the only preparation available was 11-deoxycorticosterone acetate (DOCA); it could be given IM daily or as a DOCA pellet implanted under the skin of the shoulder and it would last about 12 to 18 months. The great lesson from our study was that salt-losing patients were quite labile and very difficult to control. Indeed, they seemed to require almost daily observation with frequent adjustments to their treatment in order to maintain their salt and water balance.

At that time we found that the implantation of a DOCA pellet along with the IM cortisone acetate given every third day was the optimal treatment of these patients. Unfortunately, today the DOCA pellets and the IM cortisone acetate are no longer available (not profitable enough). Because of this, one must use *oral* preparations of cortisol or prednisolone, and fludrocortisone. This indeed makes it much more difficult to maintain electrolyte balance in those salt-losing infants.

You need to know that before the availability of cortisol and DOCA the patients with the salt-losing form of CAH would die in infancy or early childhood. We investigated the age of death: mainly they died at a few weeks of age, a few survived a few months. It was therefore quite exciting for all of us to put our data together and prepare our presentation of the promising results at the meeting of the Endocrine Society in Atlantic City.

**Figure 10:**
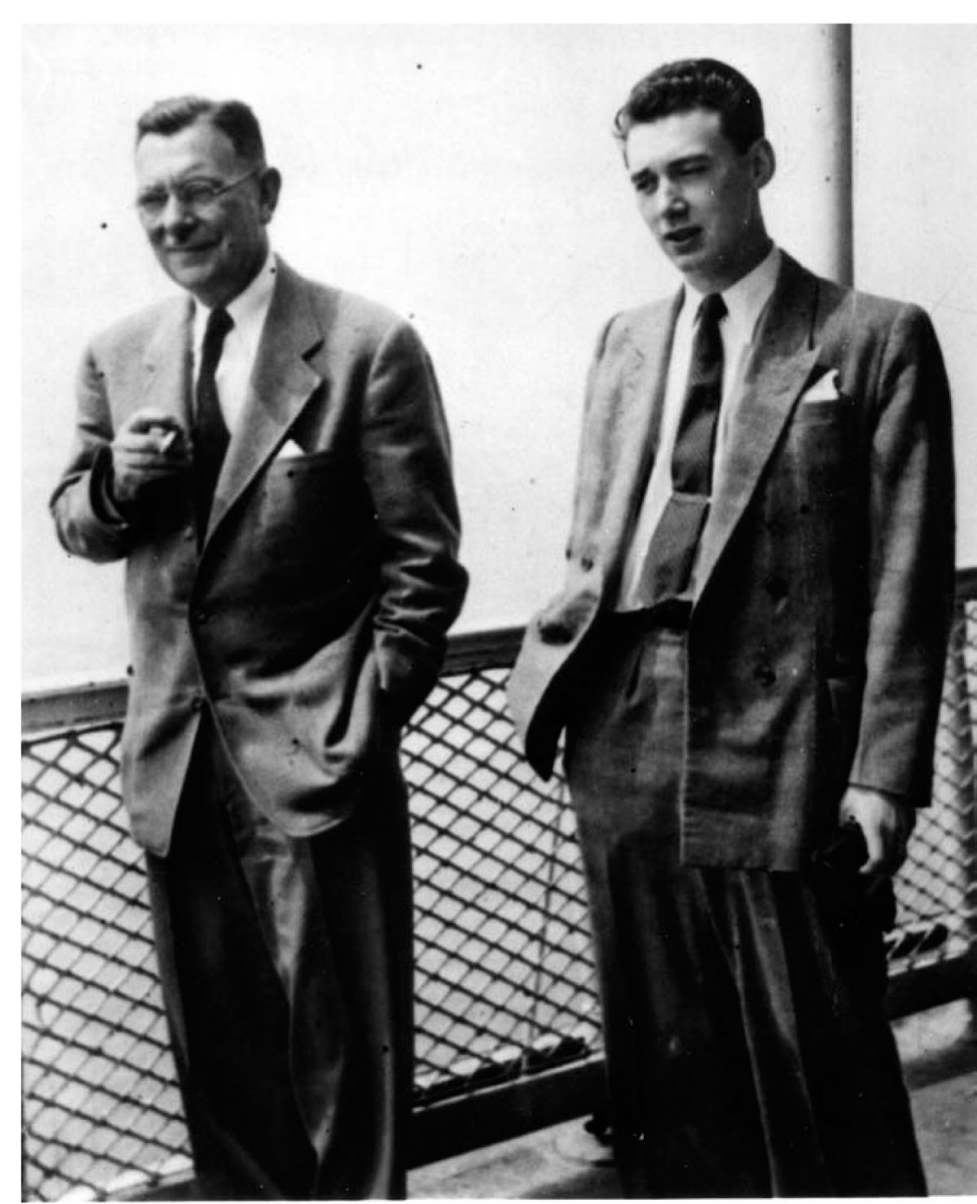
Lawson Wilkins and Claude Migeon on the ferry crossing the Susquehanna River on their way to an Atlantic City meeting. (June 1951)

A trip to Atlantic City in 1951 was an adventure. Dr. Wilkins decided that John Crigler and I would drive with him. At that time, there was no Delaware Memorial Bridge and the only way to cross the Delaware River was by ferryboat. This was not a very long voyage, but putting the car on the boat and crossing the river was quite enough excitement. This was indeed an occasion to take pictures as a memory of the voyage. (Figure 10)

Dr. Wilkins had arranged the hotel reservation; an extra bed was added to a room with twin beds so the three of us could share a very large room. During the trip, as well as in the room, Dr. Wilkins would discuss with us many of the patients whose studies would be presented at the meeting by John Crigler. The talk was extremely well-received, and the question period ran well over the allotted time. After the session, all three of us were questioned by many physicians who wanted more information and details. Of course the big wheels talked mainly to Lawson, the younger fellows talked to John and me. Needless to say, this was all very exciting. One of the physicians who asked me a question was Dr. Maria New, starting a lifelong friendship. There were also several dinners with plenty of drinks. The time for bed was usually late in the night. Then at 6:30 AM we were awakened by Dr. Wilkins who had already taken his shower and was getting dressed, as he had a meeting at 7:00 AM for some committee of the Society. He seemed as fresh as a new rose. It was amazing for me to see how much energy this man had. As for me, I had a terrible hangover from too much alcohol and too much talking the night before.

## Visit to Harvard, Massachusetts General Hospital (July 1951)

Lytt Gardner, who had come to Hopkins from Boston in July 1950, had many friends at “Mass General” where he had trained.

**Figure 11:**
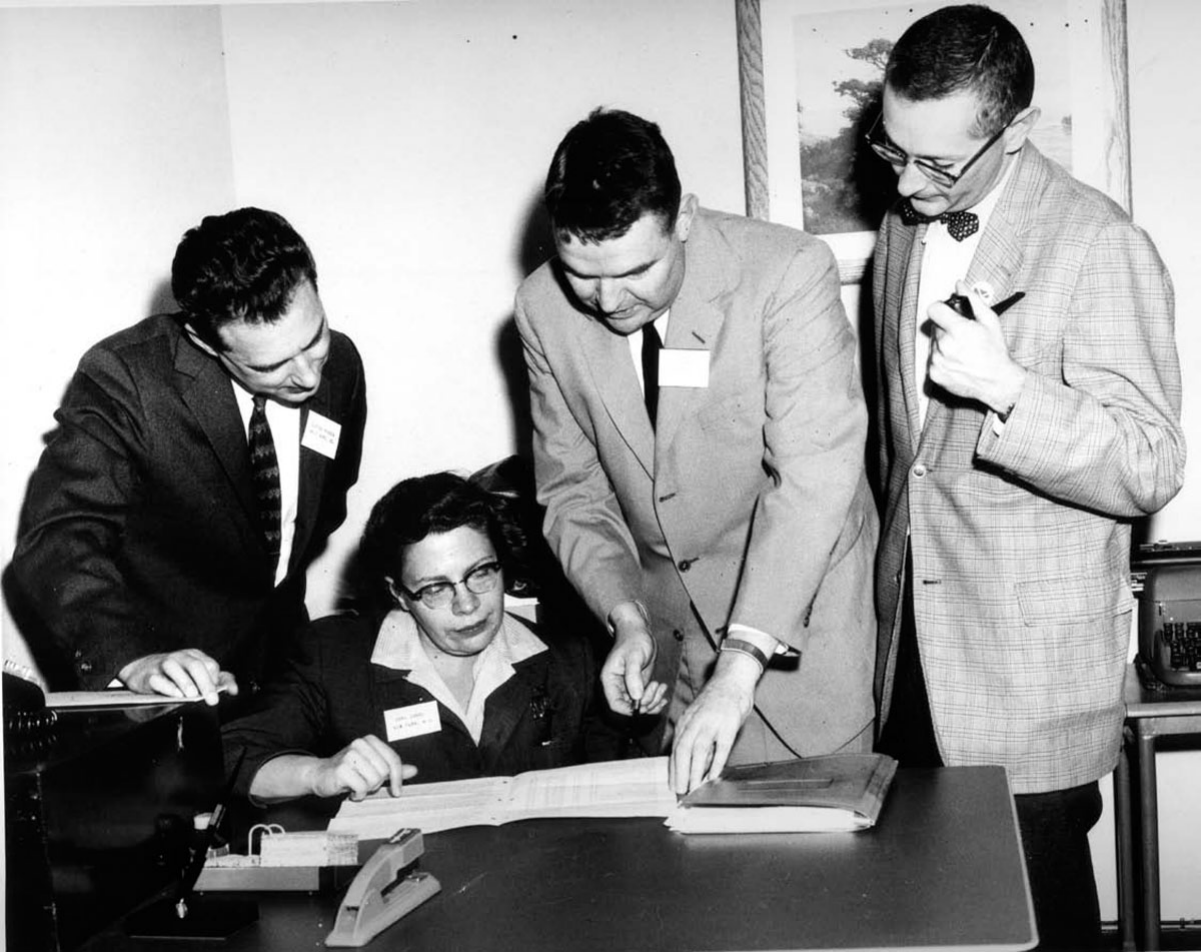
The fellows. From left: Claude Migeon, Lytt Gardner, and Robert Klein, discussing data with Edna Sobel at the Mass General Hospital in Boston. (1951)

In July 1951, he planned to visit his old colleagues and asked me to go with him. I accepted and had a lot of fun. We stayed at Lytt’s friend’s home and visited the Department of Pediatrics at Mass General. I met some of the senior physicians, Nathan Talbot and Fred Bartter, as well as some junior investigators, Jack Crawford and Edna Sobel. Lytt had been part of the group. Robert Klein also happened to be visiting from Pittsburgh Children’s Hospital. Bob had been a fellow of Wilkins from 1948-1950 with Roger Lewis (1946 – 1950) and Eugenia Rosemberg (1949 – 1950) who came from Buenos Aires. (Figure 11)

Sitting down with coffee, I was told about Fuller Albright, the most distinguished clinical scientist. He had trained in calcium metabolism with Dr. Joseph Aub. Then he came to Hopkins to work with Dr. Warfield Longcope. During that time he worked in the lab with John Eager Howard. He also spent one year with the Viennese pathologist, Jakob Erdheim.

I did not see Dr. Albright during that visit. However, the following July 1952, I was sitting near him at the Endocrine Society meeting. Despite the space between our seats, I could see the effects of Parkinson disease on this giant of endocrine science. He was shaking a great deal and had abundant secretions running from his mouth and nose. His handkerchief was hanging from his pocket but he did not seem to be able to grab it. I sat there asking myself what one should do for such an important professor in that situation. I gathered my courage and reached into his pocket, grabbed his handkerchief, and placed it in his hand. He could not talk, but his eyes seemed to say thank you. I was glad that I could help him but felt so sad that he was in such need.

## Making some beautiful music

**Figure 12:**
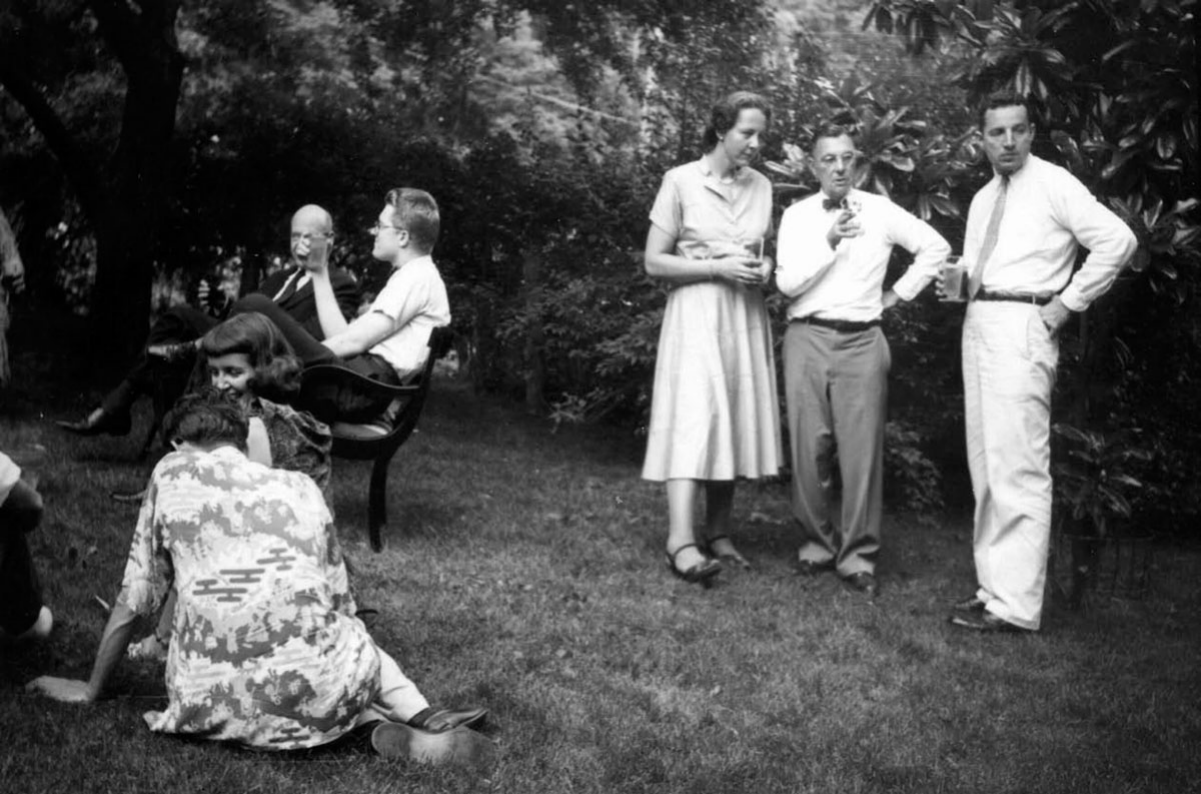
The garden parties in Wilkins’ backyard. Lawson talking with John Crigler and Janny van Walbeek of the Netherlands. (Summer 1951)

As I have previously noted, Dr. Wilkins was very gregarious and sociable. He would invite his fellows very often for an evening at his home. Some of these visits were to discuss Science but others were just for fun. It seems that Lawson had no trouble finding an excuse for a party. (Figure 12)

Whatever the nature of the party, there was always a time for singing. Lucile Wilkins would play the piano and the rest of us would sing. Lawson loved all the old English folk songs like “Go Down, Moses.” He also liked French songs, his favorite one being:

“Chevaliers de la table ronde

Gôutons voir si le vin est bon,

Gôutons voir, oui, oui, oui.

Gôutons voir, non, non, non

Gôutons voir si le vin est bon.”

Lawson particularly appreciated the “wee, wee, wee.” For this song, I was the leader of the singing group.

Anyone who has known Dr. Wilkins, must have heard him sing. Clearly, he was not great at keeping a tune, and his deep bass voice did not help the music. But, he loved to sing! So, we all sang and a little whiskey seemed to help keep the voices in tune.

At one time, Dr. Wilkins rediscovered his old violin. He told us that he had learned to play as a child and that he was practicing at night when he could not sleep. After a few months of practice, Lawson must have thought that he was ready for his entry on the music stage as he planned a soireé: he invited Dr. Harold Harrison who was a first class pianist and Dr. John Crigler who played the cello very well. There were also the regulars who made up the audience. After the three artistes had talked about the musical piece, they started playing. Quickly, something went wrong and Dr. Wilkins stopped playing. A second trial was made; just as disastrous as the first. And a third and a fourth attempt. At the fifth trial, Lawson was able to play for a short while but when the tempo accelerated, he was unable to keep up and he was begging: “Please, wait for me, wait for me!” Shortly thereafter the musical session ended. I have the feeling that it was also the end of violin playing for Lawson. But, it did not stop our singing that night, nor any night, with a glass of rye or bourbon in hand.

## Preparing to return to Paris (Summer 1952)

My travel expenses from France and my stay in Baltimore were covered by the Fulbright Foundation for 12 months at Johns Hopkins. After their year in the USA, the Fulbright Fellows were expected to return home.

Lawson Wilkins asked me about my plans and whether I would like to stay a second year. I was delighted. Then, Lawson made arrangements for me to receive support from the André and Bella Meyer Foundation and the American Friends Foundation. This was a wonderful opportunity for me as I expected the second year to be much more productive than the first one, which had included a lot of time to adapt to a new environment. And it was!

However, toward the end of my second year at Harriet Lane Home, I had to plan to return to Paris. Preliminary contacts in France suggested that my chances of continuing laboratory work and research that I had carried out at Hopkins were limited in Paris. Dr. Wilkins brought up the idea that I might consider returning to the US and that, before I left for France, I might consider my possibilities in this country. He sent my CV and his personal recommendations to several of his endocrine friends around the country, and there were four encouraging responses. Dr. Wilkins suggested that I should go for interviews with Dr. Albert Segaloff at the Alton Ochsner Foundation in New Orleans and Dr. Jack Trunnell at M.D. Anderson Hospital in Houston. A letter of Dr. Leo T. (for Tolstoy) Samuels in Salt Lake City had an offer conditional on whether Don Nelson would leave for Boston.

There was also an offer of position by Dr. Sontag, Director of the Fels Research Institute for the Study of Human Development at Antioch College.

Finally, Dr. Seymour Lieberman at Columbia, whose laboratory I had visited on several occasions, also offered me a position, sending me a telegram: “Dr. Taylor (head of gynecology) offers you Fellowship for one year beginning at your convenience.”

This number of possibilities was amazing and scary at the same time. Dr. Wilkins fully shared this excitement. We would spend time in Baltimore analyzing each offer, the site and the people involved. The discussion continued by letter (at least one a week) while I was in France.

Just before leaving Baltimore, I had supper at the Wilkins’ home and later in the evening, Lawson went through a full analysis of my long-range future. Lucile had left the two of us, alone, in the living room, Lawson standing up in front of the fireplace, his shoulders slightly vaulted, his left hand in his pocket, a cigarette in the right hand. I sat in an armchair, watching Lawson pace slowly back and forth. First, we discussed my chances to advance my career in Pediatric Endocrinology in Paris–a sub-specialty which did not exist in France at the time. Then he discussed the importance of biochemistry and physiology (at that time genetics did not dominate medical science) in biologic research. Lawson was so involved in this discussion that I had to rush with an ashtray in order to catch his long, drooping cigarette ashes. He made clear that Columbia and the University of Utah with their steroid training programs were the most advanced departments of Biochemistry in the area of steroid metabolism. Perhaps, Lawson’s personal bias made him suggest that the extensive studies of Samuels on glucocorticoids were opening new horizons in adrenal research.

That evening was probably one of the most important steps in the direction of my future career in clinical research. We had a nightcap and I went home. In present parlance, Dr. Wilkins was my “mentor.” I thought of him more as a very wise scientific father.

## Back to Paris (October – December 1952)

The trip back to France on the “Liberté” was not very comfortable as the October Atlantic Ocean was not kind. Before my departure, the goodbyes were very warm but everyone seemed to be sure that I would be back shortly. And they were right.

I found my old “chambre de bonne” at 11 Bis Rue Cesar Franck. I went to visit the family at Rethel and Lens. The prospects in Paris were not great.

I kept a running correspondence with Dr. Wilkins. I explained the pleasure of seeing my family and friends but also the lack of positions in the French medical system, as expected. I went to Zurich, to visit Andrea Prader at the Kinderspital. I had met Andrea during his visit to Baltimore. European Academic systems were quite rigid and there was no future for me in Switzerland. Meanwhile, Dr. Samuels had written to Lawson that Dr. Don Nelson had left for Boston and that he could offer me a position of Research Instructor. A specific requirement was that I should be in Salt Lake City by January 1953. Lawson let me know about it by telegram.

This was followed by a copy of Dr. Samuels’s letter which was forwarded by Lawson. On November 13, 1952, I sent a telegram and a letter of acceptance to Salt Lake City with copy to Dr. Wilkins.

On November 25, 1952, Lawson wrote:

“Dear Claude:

It was very nice to hear from you. I believe that you made the correct decision in accepting Samuels’ offer, but I hope that I did not exert too much influence on you. I realize that it is a great disappointment not to go ahead with the problem of the unknown fluorogens, but I believe that what you will learn from Samuels about corticoids and other steroids will be most profitable and that you will find him very stimulating.

I hope that you do not have any trouble about the visa. Please let me know as soon as possible your plans for returning and be sure to stop in Baltimore. It was probably difficult for you to explain your decision to your family and friends. I trust they were able to understand and accept this as the best thing for you.

Al Bongiovanni is working most enthusiastically not only on the column chromatographic pattern of the urinary steroids but also on the pregnane compounds and the blood corticosteroids. He is studying the effects of ACTH on these in adrenal hyperplasias, normals and Cushings. We hope to learn whether there is an abnormality in the synthesis of the adrenal steroids. George Clayton is measuring the conversion rate of total plasma I^131^ to PBI^131^. José and Walter are working on serum mucoproteins and urinary amino acid paper chromatography.

The only thing new to tell you is about Henry Schulte – and that is very sad. I am not sure whether you know that some years ago he had a psychiatric break and was in an institution. I did not know it until recently. Now I realize that he has been getting gradually worse for some time. He became much worse after you left and seemed completely unable to pull himself together or do anything, and all of his actions became very queer. Bongi was most kind and considerate and tried to help him, but Henry became quite paranoid and thought Bongi and others were against him. He tried to convince Mary Ellen and Betty that we were dissatisfied with him, and he went to Schwentker and everyone about his injustices, etc. Finally, on the advice of Joan Hampson and others, we decided to let him go. This was very sad.

Yours sincerely,

Lawson Wilkins, M.D.”

That was a short notice! But my telegraphic answer was an acceptance. This was followed by a frantic rush to obtain an emigration visa: Drs. Wilkins and Samuels sent letters emphasizing that I was needed for the job, and as soon as possible.

A friend from Philadelphia, Mr. Kenneth Skilling, was in Paris at that time and he helped with bank questions. The US Consulate required a Baltimore police certificate, so I wrote to Dr. Wilkins. On December 10, 1952, he answered:

“Dear Claude:

I received your letters of December 10^th^ and 12^th^, concerning the difficulties about your visa. I immediately called Mr. Frank of the Immigration and Naturalization Service. He did not know about the blank which had been sent to him, but he told me very definitely that all that you needed was a certificate from our Commissioner of Police in Baltimore stating that you had no criminal record. Fortunately, the Commissioner of Police, Col. Beverly Ober is one of my very best friends. I telephoned him about the matter, and he immediately made out the certificate which I am enclosing. I think that this will probably have to be presented at the American Consulate in Paris, and I trust it will clear up the difficulties about your visa. If there are any other problems, please let me know.

As soon as you have made plans concerning your return, please let me hear about them. You must arrange to spend some time in Baltimore. Lucile and I would like to have you stay with us, as you know we have plenty of room. Let me know when you are coming and how long you can stay.

I hope that you receive your Christmas card from us before you leave. Lucile tells me that she did not send it air mail. The Grand Marnier is magic. Under its influence, I could both speak and write French as you may see on the card.

Yours sincerely,

Lawson Wilkins, M.D.”

## Preparing for the return to the United States

After making the decision to go to Salt Lake City, I had to close my affairs in Paris. I disposed of my books and some personal items from my “chambre de bonne” by putting them in pension with my good friend Colette Bollig. I would recover them upon my return from the USA. At that point, I was still considering returning to France to spend the rest of my life, after my stay in Mormon Country. My knowledge of Mormonism at the time came from the 1921 book written by Pierre Benoit, “Le Lac Salé.” So I do not exaggerate when I say that my knowledge of Salt Lake City and its university were quite limited. Nonetheless, I looked forward to experiencing a new adventure

I said goodbye to a lot of friends. I spent Christmas with my father in Lens and New Years with my uncle and aunt who had raised me in Rethel. Then, I took the train back to Paris.

On January 10, 1953, I was on a TWA plane flying from Paris to New York with a stop in Shannon, Ireland and Reykjavik, Iceland. This was a long trip, as jet planes were not in use yet.

(Continued in Part 2, http://www.ijpeonline.com/content/2014/S1/S3, [7])

## Competing interests statement

The author has no competing interests to disclose.

## Supplementary Material

Additional file 1 Christmas 1950 menu. Click here for file

## Declarations

This article has been published as part of *International Journal of Pediatric Endocrinology* Volume 2014 Supplement 1, 2014: Remembering Doctor Lawson Wilkins: a pioneer of pediatric endocrinology. The full contents of the supplement are available online at http://www.ijpeonline.com/supplements/2014/S1.
