# Supplementary material for: Lawson Wilkins and my life: part 1
Source: Int J Pediatr Endocrinol. 2014 May 28;2014(Suppl 1):S2. doi: 10.1186/1687-9856-2014-S1-S2 (PMC4080106; doi:10.1186/1687-9856-2014-S1-S2)
Supplement: Additional file 1 — Christmas 1950 menu. [file 1687-9856-2014-S1-S2-S1.docx]

Christmas 1950

---

Pediatric Endocrine Clinic

Harriet Lane Home

Baltimore

---

Soupe á l’onion

---

Colin au Persil

*Chablis*

---

Dinde Truffée

Chataignes – Haricot Verts – Pommes Sautées

*Chateau Neuf du Pape*

---

Buche de Nöel

*Champagne Vve Cliquot*

---

Café – *Grand Marnier*
